# Longitudinal birth cohort study finds that life-course frailty associates with later-life heart size and function

**DOI:** 10.1038/s41598-021-85435-8

**Published:** 2021-03-18

**Authors:** Constantin-Cristian Topriceanu, James C. Moon, Rebecca Hardy, Nishi Chaturvedi, Alun D. Hughes, Gabriella Captur

**Affiliations:** 1 UCL MRC Unit for Lifelong Health and Ageing, University College London, Fitzrovia, London, WC1E 7HB UK; 2 UCL Institute of Cardiovascular Science, University College London, Gower Street, London, WC1E 6BT UK; 3 Cardiac MRI Unit, Barts Heart Centre, West Smithfield, London, EC1A 7BE UK; 4 CLOSER, UCL Institute of Education, 55-59 Gordon Square, London, WC1H 0NU UK; 5 Cardiology Department, The Royal Free Hospital, Centre for Inherited Heart Muscle Conditions, Pond Street, Hampstead, London, NW3 2QG UK

**Keywords:** Cardiology, Risk factors

## Abstract

A frailty index (FI) counts health deficit accumulation. Besides traditional risk factors, it is unknown whether the health deficit burden is related to the appearance of cardiovascular disease. In order to answer this question, the same multidimensional FI looking at 45-health deficits was serially calculated per participant at 4 time periods (0–16, 19–44, 45–54 and 60–64 years) using data from the 1946 Medical Research Council (MRC) British National Survey of Health and Development (NSHD)—the world’s longest running longitudinal birth cohort with continuous follow-up. From these the mean and total FI for the life-course, and the step change in deficit accumulation from one time period to another was derived. Echocardiographic data at 60–64 years provided: ejection fraction (EF), left ventricular mass indexed to body surface area (LVmassi, BSA), myocardial contraction fraction indexed to BSA (MCF_i_) and E/e′. Generalized linear models assessed the association between FIs and echocardiographic parameters after adjustment for relevant covariates. 1375 participants were included. For each single new deficit accumulated at any one of the 4 time periods, LVmass_i_ increased by 0.91–1.44% (*p* < 0.013), while MCF_i_ decreased by 0.6–1.02% (*p* < 0.05). A unit increase in FI at age 45–54 and 60–64, decreased EF by 11–12% (*p* < 0.013). A single health deficit step change occurring between 60 and 64 years and one of the earlier time periods, translated into higher odds (2.1–78.5, *p* < 0.020) of elevated LV filling pressure. Thus, the accumulation of health deficits at any time period of the life-course associates with a maladaptive cardiac phenotype in older age, dominated by myocardial hypertrophy and poorer function.

## Introduction

Cardiovascular diseases (CVDs) are the leading cause of death in the developed world and the second cause of mortality (after infectious diseases) in the developing world accounting for more than 30% of all global deaths^1^. The current knowledge base for the development of CVDs revolves mostly around traditional risk factors such as hypertension and diabetes. As such, there has been a research call for exploring novel risk factors^2^.

Given the recent medical advancements, previously deadly diseases became chronic manageable conditions. Thus, as people age, they tend to accumulate an increasing number of health deficits. However, accumulating health deficits predisposes to further deficit accumulation and premature death^3^. A frailty index quantifies the accumulated health deficits^4^ and can be appraised in either of three ways: (1) the rules-based operational definition relies on a set of phenotypical rules of performance against which the individual is scored^5^; (2) the clinical frailty score grades persons along a spectrum from very fit to very frail based on clinical judgment; and (3) the frailty index (FI) counts health deficit accumulation as the ratio between the number of deficits present, and the total number of deficits appraised, with sub-unitary scores for partial deficits^6^. The FI is most widely used in research because it is easy to score, can accommodate partial deficits, correlates with the clinical frailty score^3^, has been extensively validated and shown to predict mortality^7^ and other negative health outcomes^8^. The FI of accumulated health deficits should not be confused with a physical frailty phenotypic score that only appraises domains such as shrinking, exhaustion, low physical activity, slow gait speed and weak grip strength.

It is known that frailty is a risk factor for poor prognosis in CVDs^9^. In addition, frailty has been associated with cardiovascular mortality^10^, CVDs^11,12^ as well as with traditional CVDs risk factors such as hypertension^13^ and dyslipidemia^14^. Frailty is a state of declining physiological reserves associated with a decline in function across multiple organ systems^15^. In order to keep up with the demands of daily living, frailty has been postulated to induce a pro-catabolic, pro-oxidative and pro-inflammatory state which could predispose to CVDs^16^. However, such theory is yet to be explored using longitudinal data or in clinical studies.

To understand the association between frailty and heart size and function in older age, we analyzed longitudinal life-course data of participants from the 1946 Medical Research Council (MRC) British National Survey of Health and Development (NSHD)—the world’s longest running birth cohort with continuous follow-up. We calculated FIs at different periods of the life-course and examined their association with echocardiographic parameters at age 60–64 years.

## Methods

### Study population

Participants were from the MRC NSHD, a birth cohort study comprised of 5362 individuals born in 1 week in 1946 in Britain. The cohort has been extensively followed up with periodic assessments which have been described elsewhere^17^. Briefly, the cohort has been evaluated multi-dimensionally: anthropometrically, socio-economically, and in terms of life-style choices (e.g., smoking) and health function (e.g., mental health, cardiovascular and respiratory function).

### Ethical approval

The 2006–2010 NSHD data collection sweep included an in-depth cardiovascular assessment and was granted ethical approval from the Greater Manchester Local Research Ethics Committee and the Scotland Research Ethics Committee^17^ and written informed consent was given by all study participants. All procedures performed were in accordance with the ethical standards of the institutional and/or national research committee and with the 1964 Helsinki declaration and its later amendments or comparable ethical standards.

### Outcomes: echocardiographic parameters at 60–64 years

Between 2006 and 2010 when study members were 60–64 years, British-based NSHD participants who had not been lost to follow-up or withdrawn, were invited to attend a clinic-based assessment that included resting transthoracic echocardiography using General Electric (GE) Vivid I machines. Briefly, the echocardiographic protocol^17^ included long and short axis parasternal views, apical 5-, 4, 3- and 2-chamber views, aortic short axis view, and conventional/tissue Doppler in the apical 4-chamber. Reproducibility between readers was previously reported (intraclass correlation coefficients > 0.80)^18^. Ejection fraction (EF) was calculated by the biplane Simpson’s method. Left ventricular (LV) mass was indexed to BSA to obtain the LV mass index (LVmass_i_) as per the American Society of Echocardiography recommendations. Myocardial contraction fraction index (MCF_i_) was calculated as the ratio between stroke volume and myocardial volume. Myocardial volume was calculated by dividing LVmass_i_ by mean myocardial density (1.04 g/ml)^19^. E/e′ ratio was calculated by dividing peak mitral valve velocity (E) by the average of lateral and septal mitral annular early diastolic velocity (e′)^20^. LV end-diastolic volume index (LVEDV_i_) was derived by dividing LVEDV by BSA. Thus, our outcomes were EF, LVmass_i_, MCFi, E/e′ and LVEDV_i_.

### Exposures: frailty indices across the life-course

**Figure 1:**
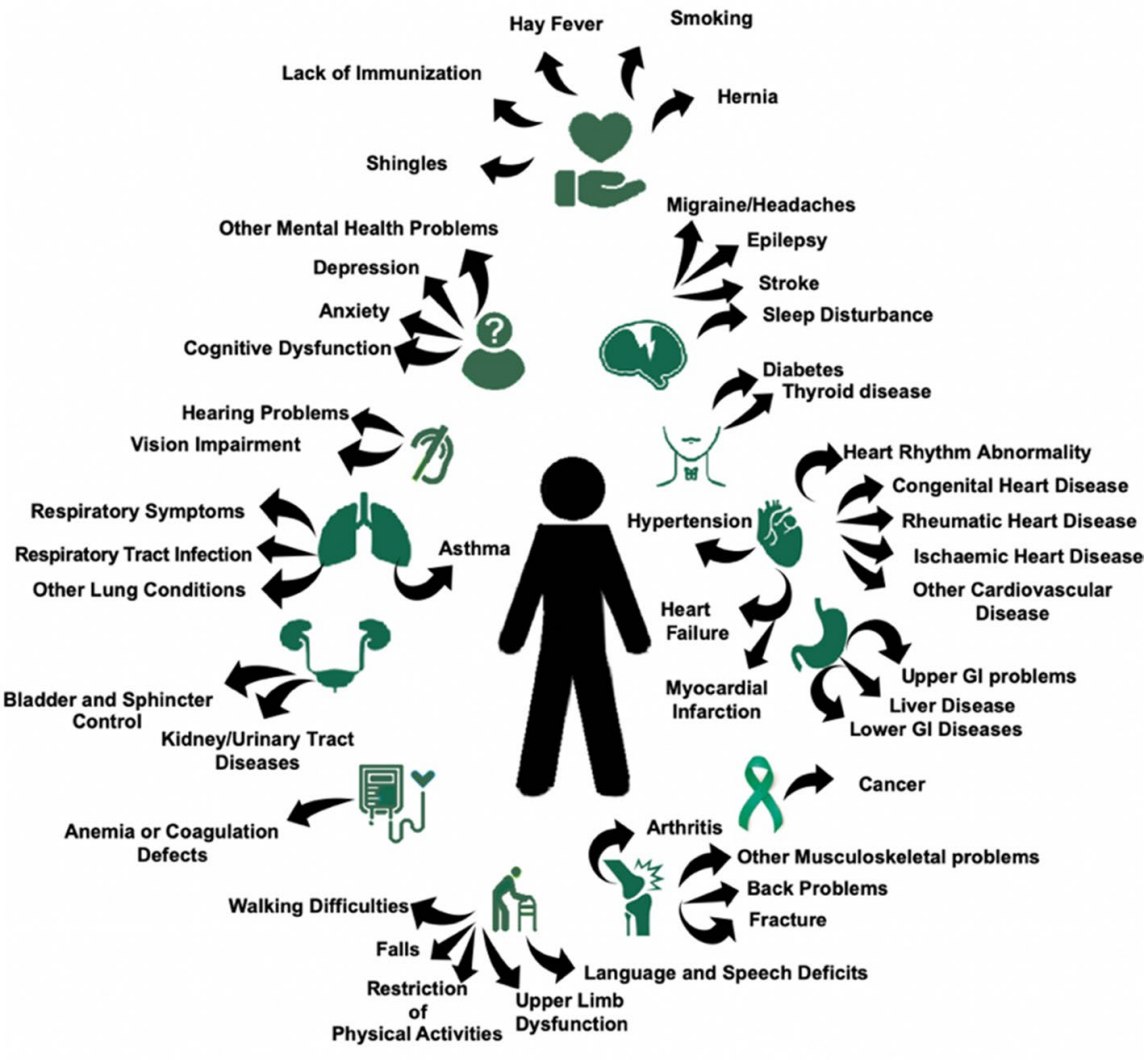
The 45 health deficit categories, spanning a broad set of physical and cognitive domains, appraised in MRC NSHD 1946 British birth cohort participants. *MRC NSHD* Medical Research Council National Survey of Health and Development, *GI* gastrointestinal.

The definition of a “health deficit” is broad and includes symptoms, signs, medical conditions, disabilities, laboratory and imaging abnormalities^4^. A “frailty index” consists of multiple “health deficits”^4^. In line with the Rockwood approach^6^ deficits included in the FI (Fig. 1, Supplementary Tables S1–S3) had to meet the following criteria: be health related; have a prevalence between 1 to 80% (i.e. neither very rare, nor very common), must not saturate early by becoming almost universal with age; must span a wide variety of medical systems; and contain less than 5% missing data^3^. To sequentially apply the same index on cohort participants, we appraised the same health deficits at each of the selected time periods of the life-course. For reliability, we aimed for an index with at least 30 health deficits in line with previous works^6^.

Health deficit score was “0” for complete absence, “1” for absolute presence, and “0.5” for partial deficits. Irreversible medical events (e.g., stroke) carried their deficit score onto all subsequent time periods, while reversible events (e.g., bone fracture) were scored de novo at each time point. To calculate the FI, the sum of health deficits present in an individual was divided by the total number of possible deficits in our index (that is 45) minus the number of missing deficits for that participant (Supplementary Eq. 1). The resultant FI can be a number from 0 (least) to 1 (most)^6^.

The time periods were selected to cover early life, young adulthood, middle age and older age. Based on the NSHD periodic follow-up assessments dates^17^, we selected the following time periods: 0–16 years, 19–44 years, 45–54 years and 60–64 years. The same frailty index (i.e., consisting of the same health deficits) has been appraised at the above four time periods resulting in four FIs across the life course: FI_0_16_, FI_19_44_, FI_45_54_ and FI_60_64_. The mathematical difference of any two of the four FI signifies the health deficits change between those time periods. Thus, to assess health deficit change between time periods, the difference between FIs at the 4 time points were computed as follows: FI_2–1_ = (FI_19_44_) − (FI_0_16_); FI_3–1_ = (FI_45_54_) − (FI_0_16_); FI_4–1_ = (FI_60_64_) − (FI_0_16_); FI_3–2_ = (FI_45_54_) − (FI_19_44_); FI_4–2_ = (FI_60_64_) − (FI_19_44_); and FI_4–3_ = (FI_60_64_) − (FI_45_54_). Lastly, to assess whole-of-life health deficit burden the four individual FIs were averaged to produce the FI_mean_ or summed to produce the FI_sum_.

Thus, three types of exposures were considered: (a) FIs at four time periods (FI_0_16_, FI_19_44_, FI_45_54_ and FI_60_64_); (b) the corresponding health deficit change between these time periods (FI_2–1_, FI_3–1_, FI_4–1_, FI_3–2_; FI_4–2_ and FI_4–3_); and (c) whole-of-life FIs (FI_mean_, FI_sum_).

### Covariates

The sex of participants was recorded as male or female. Participants’ weight and height were measured at 60–64 years and used to compute body mass index (BMI) and body surface area (BSA). Participants’ socioeconomic position (SEP) was evaluated at the time of echocardiography (60–64 years) or at 53 years where the former was not available, according to the UK Office of Population Censuses and Surveys Registrar General’s social class, dichotomized as manual or non-manual. Childhood social class has been recorded and dichotomized as above.

### Statistics

Statistical analysis was performed in R (version-3.6.3). Distribution of data were assessed on histograms and using Shapiro–Wilk test. Continuous variables are expressed as mean ± 1 standard deviation (SD) or median (interquartile range) as appropriate; categorical variables, as counts and percent. Regression models were developed using FIs or their derivatives (FI_2–1_, FI_3–1_, FI_4–1_, FI_3–2_; FI_4–2_ FI_4–3_, FI_mean_ and FI_sum_) as exposures to predict LV EF, LVmass_i_, MCF_i_ and E/e′ by echocardiography at 60–64 years as outcomes. Model 1 was adjusted for sex, Model 2 for sex and SEP, and Model 3 for sex, SEP and BMI. For LVmassi and MCFi, anthropometry was already accounted for through BSA indexation, so BMI adjustment was not additionally pursued. This decision during study design was informed through the use of directed acrylic graphs (visual representations of causal assumptions that can identify sources of confounding) that helped identify potential colliders (in this case BMI) that should be left uncontrolled, versus non-colliders (or confounders) that should be controlled in the regression models^21^.

As a result of the skewed distribution of echocardiographic parameters, generalized linear models (glm) with gamma distribution and log link were used to investigate the association of FIs with the following continuous outcome variables: LV EF, LVmass_i_ and MCF_i_; while glm with binomial distribution and logit link (equivalent to logistic regression) was employed for the binary outcome variable E/e′ using a cut-off of > 13. Although the prediction of normal and abnormal LV filling pressure is most reliable when the E/e′ is < 8 or > 15, cut-off of > 13 was chosen to avoid sensitivity loss^22^. A two-tailed *p*-value < 0.05 was considered statistically significant. Model assumptions were verified with regression diagnostics and found to be satisfied.

We ran sensitivity analyses in which we: (1) re-analyzed the associations between FIs and EF/LVmass_i_ as binary rather than continuous outcome variables using an EF cut-off of 55% and LVmass_i_ cut-offs of 102 and 88 g/m^2^ for men and women respectively, as per British Society of Echocardiography Guidelines for Chamber Quantification^23^; (2) compared the echocardiographic parameters at age 60–64 of excluded participants with > 20% missing data (for which FIs were not calculated) *vs.* included participants who had < 20% of missing data; (3) compared participants with complete echocardiographic variables and those with one or more missing echocardiographic parameter; (4) assessed whether the associations persisted if additional adjustment for childhood SEP (over and above adjustment for sex, BMI and late-adulthood SEP) was pursued, as childhood SEP can impact on adult health outcomes independently of adult SEP^24^; (5) simulated a complete case analysis in terms of the outcome variables through multiple imputation. Briefly, we have generated 50 data sets of FIs and covariates using chained equation via predictive mean matching. Regression coefficient estimates, and their associated variance metrics, were calculated for each of the 50 datasets and then combined using Rubin’s rule; (6) assessed whether the associations of FI scores with echocardiographic parameters remained unchanged if all cardiovascular-related health deficits were excluded from the FIs. Thus, we removed 8 health deficits, namely (i) hypertension, (ii) ischaemic heart disease, (iii) myocardial infarction, (iv) heart failure, (v) heart rhythm abnormality, (vi) congenital heart disease, (vii) rheumatic heart disease, and (vii) other cardiovascular diseases, from FI_0_16_, FI_19_44_, FI_45_54_ and FI_60_64_ to create FI_0_16_^-cardio^, FI_19_44_^-cardio^, FI_45_54_^-cardio^ and FI_60_64_^-cardio^ (further referred to as FIs^-cardio^); (7) assessed whether additional adjustment for childhood SEP for the FI^-cardio^ analyses affected our results; (8) simulated a complete case analysis through multiple imputation (as above) for the FI^-cardio^ analyses.

Although weighting the included health deficits limits an FI’s generalizability^25^, we performed an exploratory sensitivity analysis in which we created a weighted FI via principal component analysis (PCA) to assess the internal validity and reliability of our unweighted FI. As the first principal component typically accounts for most variability in the indicators^26^, we constructed a PCA-weighted frailty index (*wFI*) via the weighted average of the health deficits using the first principal component weights (Supplementary Table 2. This exploratory analysis was tested for two exemplar time intervals appraised by our FI that is young adulthood (19–44 years) and older age (60–64 years). For each of these *wFIs*, we then re-ran the fully adjusted regression models using *wFI*_19_44_, and *wFI*_60_64_, respectively.

In order to account for within-subject correlated repeated FIs at 0–16, 19 to 44, 45 to 54 and 60–64 years, we used random coefficients (i.e., allows each subject to have both their own intercept and their own slope in the longitudinal model) generalized linear mixed models to test the associations between the FI regarded as a longitudinal repeated measure and our echocardiographic outcomes.

To check for different profiles in the FI scores, we performed a multiple correspondence analysis (MCA) based on the indicator matrix. A scree plot was used to extract the optimal number of dimensions and to determine the percentages of inertia. From the geometric space created in the MCA, participants were then classified into clusters according to proximity criteria using the k-means algorithm. This separated participants into homogenous groups while maximizing heterogeneity across groups. This enabled us to explore whether specific clusters of participants with certain characteristics drive the associations with our echocardiographic outcomes.

## Results

### Participant characteristics and life-course burden of health deficits

Ninety health deficits were initially considered (Supplementary Table S1). Forty-five health deficits met the criteria for inclusion in the FI (Fig. 1, Supplementary Tables S2–S3). Of the 2856 participants invited to attend the clinic visit at 60–64 years, 1690 attended and 1653 had echocardiography of which 1617 had acceptable image quality. Of these, 1375 study members had < 20% missing data and at least one outcome parameter of interest by echocardiography. Out of the 1375 NSHD participants studied, 1 had an FI = 0 plus missing health deficit data of up to 20%, while 3 had FI = 0 in the absence of missing data.

Participant characteristics and FI results are summarized in Table 1. Generally, higher FI_sum_ and FI_mean_ were associated with female sex, lower SEP and higher BMI. For the vast majority of participants FI increased across the life-course, but in 86 participants (0.05%) FI decreased in spite of aging, largely due to the reversible nature of some health deficits. There was no significant difference in echocardiographic parameters between participants whose life-course FIs decreased when compared to the rest of the cohort (LV EF *p* = 0.30, LVmass_i_ *p* = 0.99, MCF_i_ *p* = 0.08, E/e′ *p* = 0.57).

**Table 1:** Characteristics of the MRC NSHD sample considering participants with less than < 20% missing health deficit data and having at least one echocardiographic parameter of interest (EF, MCF_i_, LVmass_i_ or E/e′).

	Overall		Men		Women	
	*n*	Result	*n*	Result	*n*	Result
Age, years	1375	63.2 ± 1.1	639	63.2 ± 1.2	736	63.3 ± 1.1
Height, m	1375	1.68 ± 0.88	639	1.75 ± 0.06	736	1.62 ± 0.06
Weight, kg	1375	78.13 ± 14.80	639	84.60 ± 12.79	736	73.16 ± 14.66
BMI	1375	27.62 ± 4.63	639	27.63 ± 3.91	736	28.03 ± 5.59
SEP non-manual^a^	1375	1003 (72.94)	639	436 (63.31)	736	567 (77.04)
SEP manual^b^	1375	372 (31.76)	639	203 (31.77)	736	169 (22.96)
LVEDV_i_	1222	48.25 ± 11.18	579	52.18 ± 12.6	643	44.72 ± 8.27
IVSD, cm	1256	1.06 ± 0.25	581	1.12 ± 0.24	675	1.01 ± 0.24
PWT, cm	1254	0.99 ± 0.21	580	1.06 ± 0.24	674	0.94 ± 0.17
RWT	1254	0.44 ± 0.11	580	0.44 ± 0.11	674	0.43 ± 0.11
LAVI, ml/m^2^	1195	22.08 ± 7.59	571	23.13 ± 7.90	624	21.12 ± 7.16
EF, %	1217	64.41 ± 7.89	576	63.45 ± 7.07	640	65.28 ± 7.63
LVmass_i_, g/m^2^	1006	114.91 ± 37.50	448	129.38 ± 41.30	558	103.30 ± 29.39
MCF_i_	860	0.49 ± 0.19	395	0.52 ± 0.20	465	0.48 ± 0.18
E/e′	1259	7.95 ± 2.13	569	7.51 ± 2.03	690	8.3 ± 2.15
FI_0_16_	1375	0.13 (0.11, 0.17)	639	0.13 (0.10, 0.16)	736	0.14 (0.11, 0.17)
FI_19_44_	1375	0.13 (0.09, 0.18)	639	0.13 (0.09, 0.17)	736	0.15 (0.09, 0.19)
FI_45_54_	1375	0.21 (0.15, 0.27)	639	0.17 (0.14, 0.22)	736	0.23 (0.18, 0.29)
FI_60_64_	1375	0.27 (0.21, 0.33)	639	0.24 (0.19, 0.30)	736	0.29 (0.23, 0.36)
FI_2–1_	1375	0.00 (-0.04, 0.04)	639	-0.01 (-0.05, 0.03)	736	0.01 (-0.04, 0.06)
FI_3–1_	1375	0.07 (0.02, 0.13)	639	0.04 (-0.01, 0.09)	736	0.10 (0.05, 0.16)
FI_4–1_	1375	0.13 (0.07, 0.20)	639	0.11 (0.06, 0.17)	736	0.15 (0.09, 0.21)
FI_3–2_	1375	0.07 (0.03, 0.11)	639	0.05 (0.02, 0.08)	736	0.09 (0.05, 0.13)
FI_4–2_	1375	0.13 (0.09, 0.18)	639	0.12 (0.08, 0.16)	736	0.14 (0.09, 0.20)
FI_4–3_	1375	0.06 (0.02, 0.10)	639	0.06 (0.03, 0.10)	736	0.05 (0.01, 0.09)
FI_sum_	1375	0.74 (0.59, 0.91)	639	0.67 (0.56, 0.83)	736	0.81 (0.65, 0.98)
FI_mean_	1375	0.19 (0.15, 0.23)	639	0.17 (0.14, 0.21)	736	0.20 (0.16, 0.25)

Data presented as mean ± standard deviation, median (interquartile range) or counts (%) as appropriate.

*BMI* body mass index, *EF* ejection fraction, *FI*_*0_16*_ frailty index at 0–16 years, *FI*_*19_44*_ frailty index at 19–44 years, *FI*_*45_54*_ frailty index at 45–54 years, *FI*_*60_64*_ frailty index at 60–64 years, *FI*_*2–1*_ step change in deficit accumulation between FI_19_44_ and FI_0_16_, *FI*_*3–1*_ step change in deficit accumulation between FI_45_54_ and FI_0_16_, *FI*_*4–1*_ step change in deficit accumulation between FI_60_64_ and FI_0_16_, *FI*_*3–2*_ step change in deficit accumulation between FI_45_54_ and FI_19_44_, *FI*_*4–2*_ step change in deficit accumulation between FI_60_64_ and FI_19_44_, *FI*_*4–3*_ step change in deficit accumulation between FI_60_64_ and FI_45_54_, *FI*_*sum*_ sum of FI_0_16_, FI_19_44_, FI_45_54_ and FI_60_64_, *FI*_*mean*_ mean of FI_0_16_, FI_19_44_, FI_45_54_ and FI_60_64_, *IVSD* interventricular septal thickness in diastole, *LAVI* left atrial volume indexed to body surface area, *LVEDV*_*I*_ left ventricular end-diastolic volume indexed by the body surface area, *LVmassi* left ventricular mass indexed to body surface area, *MCF*_*i*_ myocardial contraction fraction indexed to body surface area, *PWT* left ventricular posterior wall thickness in diastole, *RWT* relative wall thickness, *SEP* socio-economic position.

^a^Defined as SEP classes I–IIIN.

^b^Defined as SEP classes IIIM–V.

**Figure 2:**
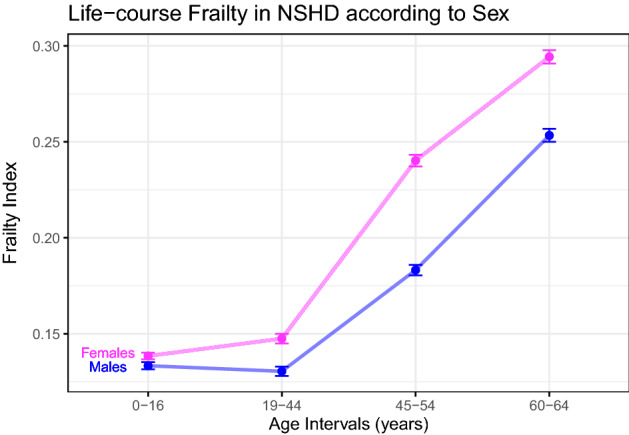
Sex differences in life-course burden of health deficits in the NSHD cohort. Females (pink) had higher FIs (*y*-axis) than males (blue) at every time period (*x*-axis). Vertical bars represent standard error of the means. Abbreviations as in Fig. 1.

There was no difference in FI between the ages 0–16 and 19–44 years, but increases were observed between young adulthood and early middle age, and between early middle age and older age. Raw FI scores of NSHD participants are displayed in Supplementary Fig. S1. Health deficit contributors at the 4 time periods are shown in Supplementary Fig. S2. Women consistently had higher FIs than men at every age interval (Fig. 2).

The association between FIs and echocardiographic parameters are presented in Table 2. For each of our echocardiographic outcomes, we present the associations from two perspectives: (i) if there is a unit increase in the FI (i.e., from 0 to 1) which means accumulating all the 45 health deficits of the index and (ii) if only 1 out of the 45 health deficits (any of them) is gained which translates into a 0.02 increase in FI. The fully adjusted model refers to the model where we have adjusted for sex, SEP and BMI (the latter excluded if the echocardiographic parameter was already indexed to BSA).

**Table 2:** Associations between health deficit burden at the 4 time periods and whole-of-life health deficit burden, with echocardiographic parameters (EF, LVmass_i_, MCF_i_ and E/e′) at 60–64 years.

Echo parameter^a^	FI	Model 1 (adjusted for sex)		Model 2 (adjusted for sex + SEP)		Model 3 (adjusted for sex + SEP + BMI)	
		*β* (95% CI)	*p*-value	*β* (95% CI)	*p*-value	*β* (95% CI)	*p*-value
EF	FI_0_16_	0.03 (− 0.12, 0.18)	0.667	0.03 (− 0.12, 0.17)	0.745	0.04 (− 0.11, 0.19)	0.623
	FI_19_44_	− 0.10 (− 0.20, 0.00)	0.058	− 0.11 (− 0.21, 0.00)	0.055	− 0.10 (0.20, 0.01)	0.070
	FI_45_54_	− 0.12 (− 0.21, − 0.03)	**0.010**	− 0.12 (− 0.21, − 0.03)	**0.007**	− 0.12 (− 0.21, − 0.03)	**0.013**
	FI_60_64_	− 0.12 (− 0.20, − 0.05)	**0.001**	− 0.13 (− 0.21, − 0.06)	**< 0.001**	− 0.13 (− 0.20, − 0.05)	**0.002**
	FI_sum_	− 0.04 (− 0.07, − 0.01)	**0.009**	− 0.04 (− 0.07, − 0.01)	**0.006**	− 0.04 (− 0.07, − 0.01)	**0.011**
	FI_mean_	− 0.16 (− 0.28, − 0.04)	**0.009**	− 0.17 (− 0.29, − 0.05)	**0.006**	− 0.16 (− 0.28, − 0.04)	**0.011**
LVmass_i_	FI_0**_**16_	0.58 (0.18, 0.97)	**0.005**	0.50 (0.11, 0.90)	**0.013**		
	FI_19_44_	0.46 (0.19, 0.74)	**0.001**	0.41 (0.14, 0.69)	**0.003**		
	FI_45_54_	0.40 90.16, 0.63)	**< 0.001**	0.34 (0.11, 0.58)	**0.004**		
	FI_60_64_	0.40 (0.20, 0.61)	**< 0.0001**	0.36 (0.16, 0.56)	**< 0.001**		
	FI_sum_	0.17 (0.09, 0.25)	**< 0.0001**	0.15 (0.07, 0.23)	**< 0.001**		
	FI_mean_	0.67 (0.36, 0.99)	**< 0.0001**	0.60 (0.28, 0.91)	**< 0.001**		
MCF_i_	FI_0_16_	− 0.66 (− 1.23, 0.09)	**0.023**	− 0.61 (− 1.18, − 0.03)	**0.039**		
	FI_19_44_	− 0.53 (− 0.91, − 0.14)	**0.008**	− 0.48 (− 0.87, − 0.09)	**0.016**		
	FI_45_54_	− 0.38 (− 0.69, − 0.06)	**0.025**	− 0.32 (− 0.65, − 0.001)	0.054		
	FI_60_64_	− 0.43 (− 0.70, − 0.15)	**0.003**	− 0.38 (− 0.66, − 0.10)	**0.009**		
	FI_sum_	− 0.18 (− 0.29, − 0.07)	**0.002**	− 0.16 (− 0.27, − 0.05)	**0.006**		
	FI_mean_	− 0.71 (− 1.14, − 0.27)	**0.002**	− 0.63 (− 1.07, − 0.19)	**0.006**		
E/e′	FI_0_16_	4.70 (-3.27, 12.39)	0.239	4.76 (− 3.22, 12.45)	0.234	2.72 (− 5.31, 10.72)	0.489
	FI_19_44_	0.77 (− 5.01, 6.22)	0.788	0.81 (− 4.99, 6.28)	0.778	− 0.82 (− 6.83, 4.84)	0.782
	FI_45_54_	4.28 (− 0.35, 8.65)	0.062	4.39 (− 0.28, 8.82)	0.058	2.87 (− 1.99, 7.49)	0.235
	FI_60_64_	6.39 (2.62, 10.06)	**< 0.001**	6.50 (2.71, 10.18)	**< 0.001**	5.26 (1.37, 9.06)	**0.007**
	FI_sum_	1.78 (0.23, 3.27)	**0.021**	1.82 (0.26, 3.33)	**0.019**	1.26 (− 0.36, 2.83)	0.119
	FI_mean_	7.12 (0.91, 13.07)	**0.021**	7.30 (1.04, 13.31)	**0.019**	5.05 (− 1.45, 11.31)	0.119

*β* regression coefficient, *CI* confidence interval. Other abbreviations as in Table 1.

^a^All reported analyses here consisted of generalized linear models with gamma Distribution and log link except for E/e′ that used logistic regression. Significant *p*-values are highlighted in bold.

### Ejection fraction

**Figure 3:**
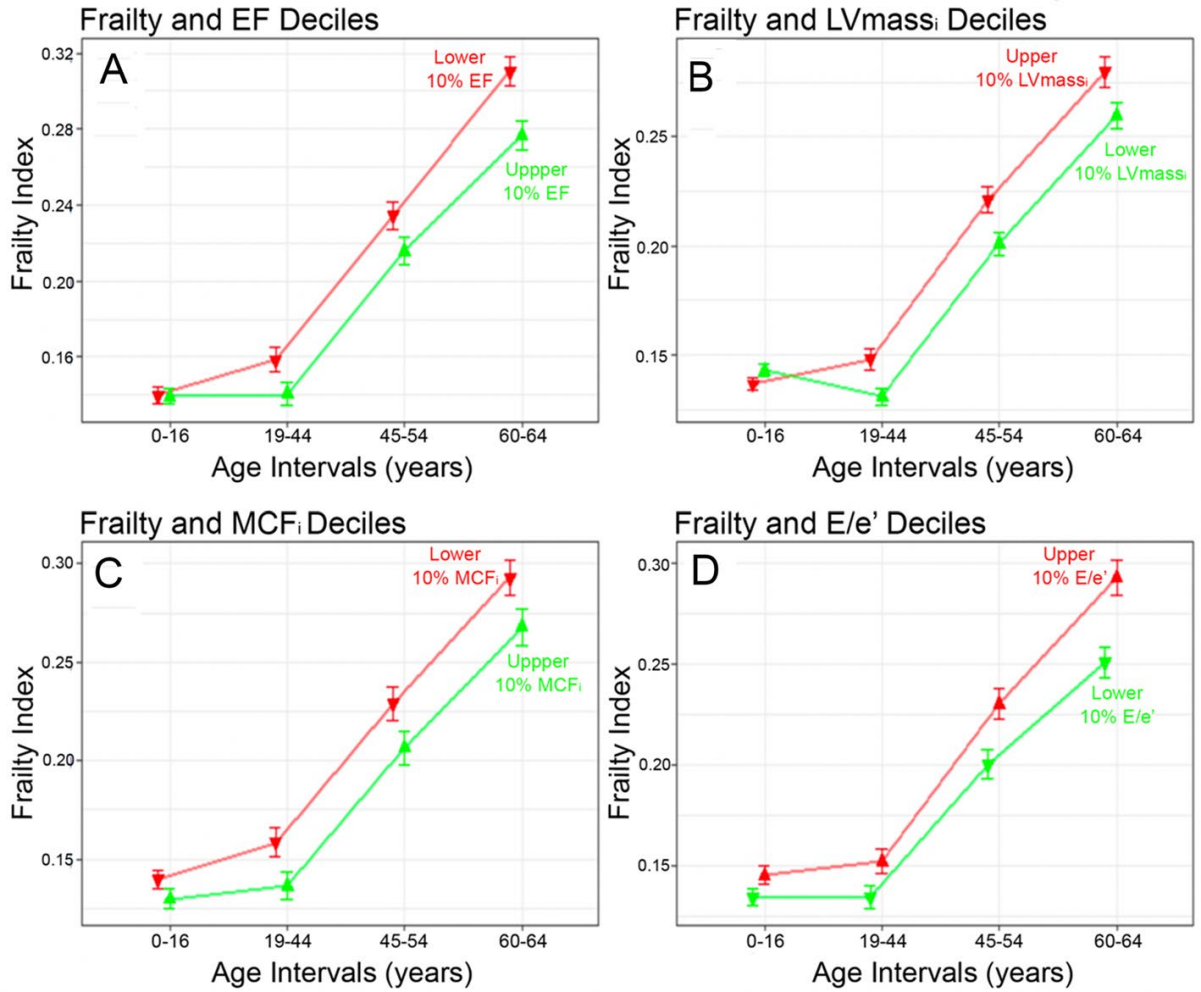
Comparing trajectories of mean life-course FI (*y*-axis) at each age interval (*x*-axis) in terms of the older age echocardiographic phenotype. FI trajectories leading to favourable phenotypes shown in green and unfavourable trajectories in red as follows: (**A**) highest (green) vs. lowest (red) LV EF deciles; (**B**) highest (red) vs. lowest (green) LVmass_i_ deciles; (**C**) highest (green) vs. lowest (red) MCF_i_ deciles; and (**D**) highest (red) vs. lowest (green) E/e′ deciles. Vertical bars represent standard error of the means. Wilcoxon signed-rank test *p*-values (not shown) for median inter-decile Differences were < 0.05 at every time point except for the 0–16 years age interval. *EF* ejection fraction, *LVmass*_*i*_ left ventricular mass index, *MCF*_*i*_ myocardial contraction fraction index. Other abbreviations as in Fig. 1.

In fully adjusted models, a unit increase in FI in early middle age (FI_45_54_) or older age (FI_60_64_) translated into an 11–12% decrease in EF (*p* = 0.013 and 0.002). Thus, accumulating a single health deficit associated with a 0.24–0.27% in EF. A unit increase in FI_sum_ and FI_mean_ translated into a 4–15% decrease in EF (*p* = 0.011 both). When comparing the FIs of participants with the highest and lowest deciles of EF, those with the lowest EF had higher FIs throughout life except in early life (0–16 years) (Fig. 3A).

### Left ventricular mass index

In fully adjusted models, a unit increase in FI translated into a 16–82% increase in LVmass_i_ (all p < 0.013). The accumulation of a single new health deficit in early-life, young adulthood, early middle age or older age, led to a 1.44%, 1.13%, 0.91% and 0.96% increase in LVmass_i_ respectively. The group of participants with the highest decile of LVmass_i_ had significantly higher FIs throughout life when compared to the group with the lowest decile, except in early-life (Fig. 3B).

### Myocardial contraction fraction index

In fully adjusted models, a unit increase in FI translated into a 15–47% decrease in MCF_i_ (all *p* < 0.05). The accumulation of a single new health deficit in early-life, young adulthood, early middle age or older age, led to a 1.02%, 0.85%, 0.60% and 0.71% decrease in MCF_i_ respectively. When comparing the FIs of participants with the highest and lowest deciles of MCF_i_, those with the lowest MCF_i_ had significantly higher FIs throughout life except in early-life (Fig. 3C).

### E/e′ ratio

A unit increase in FI in older age increased the log odds of having elevated LV filling pressure (E/e′ > 13) by 5.3 (*p* = 0.007) in fully adjusted models. The acquisition of new health deficits at any time between early-life to older age increased the multiplicative odds of having elevated LV filling pressure in older age by 2.1 times for FI_4–1_, by 75.4 times for FI_4–2_ and 78.5 times for FI_4–3_ (Supplementary Table S4). Participants with the highest decile of E/e′ had significantly higher FIs throughout life when compared to those in the lowest decile (Fig. 3D).

### LVEDV_i_

After adjustment for sex and SEP there was no association between any of the FIs and LVEDV_i_ (Supplementary Table S5).

### Sensitivity analysis

There was no significant difference (*p* > 0.105) between the echocardiographic parameters of participants included in the FI analysis compared to those excluded due to > 20% missing data (Supplementary Table S6). There was no significant difference in cardiac values between those with complete or some missing echocardiographic parameters (Supplementary Table S7). After additional adjustment for childhood SEP, *β* coefficients were slightly attenuated but FIs mostly retained significant associations with echocardiographic parameters (Supplementary Table S8). Associations between FIs and echocardiographic parameters persisted in fully adjusted models after multiple imputation (Supplementary Table S9). After removing cardiovascular health deficits (we removed hypertension, ischaemic heart disease, myocardial infarction, heart failure, heart rhythm abnormality, congenital heart disease, rheumatic heart disease, and other cardiovascular diseases) from all the FIs, associations of early-life and young adulthood health deficit burden with the 4 key echocardiographic parameters remained significant for LVmass_i_ and MCF_i_ (Supplementary Table S10). Associations for FIs^-cardio^ persisted even further adjustment for childhood SEP (Supplementary Table S11), but the regression coefficients were slightly attenuated. Similarly, the associations for FIs^-cardio^ mostly remained after multiple imputation (Supplementary Table S12). Associations between PCA *wFIs* and LVmass_i_, and MCF_i_ persisted both in young adulthood and older age, but were lost for EF and E/e′ (Supplementary Table S13). Associations between the FI regarded as a longitudinal variable repeatedly measured at 4 data points (i.e., the time periods 0–16 years, 19–44 years, 45–54 years and 60–64 years) and EF, LVmass_i_ and MCF_i_ persisted when using random coefficients generalized linear mixed models (Supplementary Table S14).

The cluster analysis revealed no clear separation of participants in terms of health deficit patterns (from the 45 health deficits in our index) in any of four age-intervals appraised (0–16, 19–44, 45–54 and 60–64 years) (Supplementary Figs. S3–S6).

## Discussion

Life-course data from the MRC NSHD British birth cohort indicates that health deficits accumulated during childhood, young adulthood, middle age and older age, and the accumulation of new deficits between these periods, associate with an adverse cardiac phenotype in older age—one that is characterized by hypertrophy, elevated filling pressure and reduced systolic function.

A frailty index objectively captures the vulnerability of an individual by counting health deficits. Although theoretically a FI can have any value from 0 to 1, values higher than 0.5 were rare amongst NSHD participants (< 1%), as a high burden of health deficits limits survival^27^.

The burden of health deficits in all 4 time periods of the life-course, including early-life and young adulthood, was associated with higher LVmass_i_ and lower MCF_i_ in later-life. The accumulation of a single health deficit at any time period of the life-course (0–16 years, 19–44 years, 45–54 years and 60–64 years) potentially results in a 0.9–1.4% increase in LVmass_i_ and a 0.6–1% decrease in MCF_i_ at 60–64 years. A possible explanation may be that health deficits insidiously strain the heart which eventually manifests as maladaptive myocardial hypertrophy. Even after removing all cardiovascular-related health deficits from the FIs, the associations remained significant, particularly in early-life, confirming the biological relationship linking any kind of global health deficits with cardiac function parameters in older age.

We found that NSHD participants with decreasing FIs across the life-course, had fitter hearts when compared to the rest of the cohort. Recognizing the potential long-term cardiovascular sequelae of health deficit accrual in these early formative years opens a window of opportunity for managing health-deficits earlier rather than later. This means firmer implementation of public health strategies aimed at disease prevention, the integrated treatment of conditions as soon as they arise, and provision of more holistic and integrated models of healthcare to ensure that optimal care is provided for all co-existing conditions. Importantly, there is a need to ensure that the priorities and preferences of patients are integrated in all of these strategies to maximize what may well become their life-course engagement with the healthcare system.

Whole-of-life burden of health deficits (FI_sum_ and FI_mean_) was associated with lower EF and MCF_i_, and higher LVmass_i_ in later-life, more so for FI_mean_. The latter is likely due to the over-inflation of the FI brought about by summation (in FI_sum_) which absorbs all health deficits including reversible ones. Whole-of-life FIs were not significantly associated with elevated LV filling pressures, but the degree of health deficit accumulation between FI_60_64_ and any of the preceding time periods (FI_4–1_, FI_4–2_ and FI_4–3_) were. The acquisition of new health deficits at any time between early-life to older age (FI_4–1_) increased the multiplicative odds of having elevated LV filling pressure in older age by 2.1. If new health deficits were acquired later, the multiplicative odds of having elevated LV filling pressure in older age were much higher (75.4 times for FI_4–2_ and 78.5 times for FI_4–3_). In a similar way, these step changes in health deficit accumulation, but also those occurring earlier in life (FI_2–1_, FI_3–1_) were associated with lower LV systolic function at 60–64. These data suggest that regardless of one’s baseline health deficit burden, accumulation of multiple health deficits increases one’s risk of cardiac dysfunction in the senior years. It reinforces the point that even apparently mild de novo clinical issues should be taken seriously, to prevent them from having a long-term destabilizing effect on the myocardium. This is especially important given that with modern medicine, people are living longer, and previously deadly diseases are now being turned into chronic manageable problems contributing to multi-morbidity. It is already well-established that multi-morbidity erodes our well-being and quality of life, predisposing to further health deficit accumulation, premature death, mental health problems, negative health behaviours and reduced self-care conduct^28^. What the current work adds to the multi-morbidity knowledgebase, is this notion that multiple health deficit accumulation is harmful for the heart.

Frailty has been previously associated with heart failure^29^. Heart failure with preserved ejection fraction (HFpEF) is more common than heart failure with reduced ejection fraction (HFrEF) in frail individuals^30^. Our results show that a higher frailty index in senior years as well as health deficit accumulation throughout the life-course is consistent with both. As there were only 4 individuals with concurrent HFpEF (as defined by an E/e′ > 13) and HFrEF (as defined by EF < 55%), we present mostly non-overlapping effects (as there were 24 with sole HFpEF and 116 with sole HFrEF). Speculatively, different patterns or different rates of health deficit accumulation could strain the organism in different ways paving the way to either HFpEF or HFrEF. This highlights that health deficit burden is more complex than previously postulated and future research should concentrate on exploring the longitudinal effects of clusters of accumulated health deficits. In addition, the prospect of incorporating the frailty index in scores predicting HF should also be evaluated.

A strength of the study is the implicit age homogeneity of birth cohort participants enabling age-matching at all subsequent assessments for FIs. Participants were exposed to similar risk factors and had access to similar diagnostic and treatment facilities over time. However, such secular effects may also have led to the under-estimation of early-life FIs through underdiagnoses. Conversely, the burden of certain medical conditions may have been over-represented in early-life FIs compared to a modern-day population because treatments exist now that may not have been available then. Another strength of our work is the relatively large FI (i.e., 45-items) which spans multiple health domains, as it has been well established that the biggest predictors for an FI’s performance are its size and broadness of deficits covered^6,31^. Traditionally, it was believed that the equal weighting assigned to heterogenous health deficits (e.g., reversible and irreversible, minor and major etc.) that are unlikely to be straining the organism in a commensurate way was a limitation of quantitative FIs. However, the current knowledge perceives it as strength^25^, as although weighting of the deficits improves the model fit, it limits the generalizability. Regardless, we performed a PCA and showed that the associations persisted for both LVmass_i_, and MCF_i_, even when the frailty index was formulated as a weighted average of health deficits. The associations were lost for EF and E/e′ because the weighted index diluted the contributions of cardiovascular deficits upon which EF and E/e’ strongly rely, as previously revealed by the sensitivity analysis (Supplementary Table S10). However, PCA loading on the first principal component can make the weighting contributions of health deficits specious. As the health deficits were scored over relatively large time periods, the presence of random zero FIs was negligibly low in the analyzed sample. Thus, a strength of our data is the absence of zero-inflation and overdispersion.

As some health deficits included in our frailty index were self-reported, misclassification bias could result in FI inflation or deflation. In addition, we did not account for the possibility of some health deficits spilling over (through causation or modulation) into other conditions. Modelling longitudinal data to derive the time periods wherein to appraise our frailty index would have been ideal, but it was not possible due to the periodic nature of the NSHD follow-up assessments at specific dates where specific health variables were evaluated. The timing of echocardiographic assessment in the 7th decade of life excludes participants that already passed away who might have had the highest FIs and most adverse cardiac phenotype. Moreover, selective follow-up could also be a potential confounder as changes in LV morphology could be a protective mechanism against mortality. There are recognized precision limitations in the GE Vivid I system used to capture all echocardiographic data in this study. Whilst the current data indicate a significant association between health deficit burden and echocardiographic parameters at 60–64 years, the evidence is insufficient to claim a causal line. Despite the association between high E/e′ and elevated LV filling pressure, there are instances where it is unreliable^32^.

Frailty has been previously associated with CVDs^11,12^. However, the directionality of the association is still a matter of debate. As previous studies indicated an association between frailty and CVDs risk factors such as hypertension^13^, the theory that frailty predisposes to CVDs has emerged^16^. Using longitudinal data from the world’s longest running birth cohort with continuous follow-up, we have shown that accumulation of health deficits across the life course associates with later life heart dysfunction. Although our study design can’t establish causality, we provide insight into the potential cardiac consequences of health deficit accumulation.

## Conclusion

The accumulation of health deficits during the life-course associates with a maladaptive cardiac phenotype in older age characterized by hypertrophy and poorer function. It could be that health deficits accumulated during childhood, young adulthood, middle age and older age, insidiously strain the myocardium potentially paving the way to future cardiac dysfunction in susceptible individuals.

## Supplementary Information

Supplementary Information.

## Data Availability

NSHD data is available from: https://www.nshd.mrc.ac.uk/data.
