# Supplementary material for: Longitudinal birth cohort study finds that life-course frailty associates with later-life heart size and function
Source: Sci Rep. 2021 Mar 18;11:6272. doi: 10.1038/s41598-021-85435-8 (PMC7973558; doi:10.1038/s41598-021-85435-8)

**Supplementary Material for:**

**Longitudinal birth cohort study finds that life-course frailty associates with later-life heart size and function**

Constantin-Cristian Topriceanu1, James C Moon2,3, Rebecca Hardy4, Nish Chaturvedi1,2, Alun D Hughes1,2, Gabriella Captur1,2,5

**Author Affiliations:**

1. UCL MRC Unit for Lifelong Health and Ageing, University College London, Fitzrovia, London WC1E 7HB
2. UCL Institute of Cardiovascular Science, University College London, Gower Street, London WC1E 6BT, UK
3. Cardiac MRI Unit, Barts Heart Centre, West Smithfield, London, EC1A 7BE
4. CLOSER, UCL Institute of Education, 55-59 Gordon Square, London WC1H 0NU
5. The Royal Free Hospital, Centre for Inherited Heart Muscle Conditions, Cardiology Department, Pond Street, Hampstead, London NW3 2QG, UK

**Supplementary Equation 1 1**: Formula for calculating the FI.


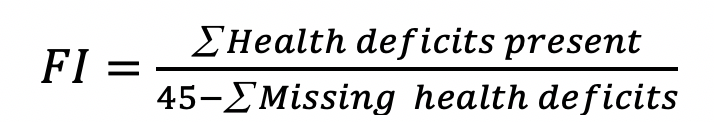


**Supplementary Table S1.** Comprehensive list of considered health deficit candidates.

| **Frailty index: Deficit Candidates**  **(n=90)** | **Medical System Categories**  **(n=15)** | **Inclusion status*** |
| --- | --- | --- |
| Congenital heart disease | Cardiovascular system | Included |
| Rheumatic heart disease | Cardiovascular system | Included |
| Hypertension | Cardiovascular system | Included |
| Ischaemic heart disease | Cardiovascular system | Included |
| Myocardial infarction | Cardiovascular system | Included |
| Heart failure | Cardiovascular system | Included |
| Heart rhythm abnormality | Cardiovascular system | Included |
| Cardiomyopathy | Cardiovascular system | Excluded |
| Valvular heart disease | Cardiovascular system | Excluded |
| Peripheral artery disease | Cardiovascular system | Excluded |
| Other cardiovascular disease | Cardiovascular system | Included |
| Anemia | Haematological system | Excluded |
| Clotting disorders | Haematological system | Excluded |
| Anemia or Clotting disorders | Haematological system | Included |
| Diabetes | Endocrine system | Included |
| Thyroid disease | Endocrine system | Included |
| Other Endocrine Disease | Endocrine system | Included |
| Asthma | Respiratory system | Included |
| Chronic obstructive pulmonary disease | Respiratory system | Excluded |
| Bronchiectasis | Respiratory system | Excluded |
| Interstitial lung disease | Respiratory system | Excluded |
| Obstructive sleep apnea | Respiratory system | Excluded |
| Respiratory Tract Infection | Respiratory system | Included |
| Respiratory symptoms | Respiratory system | Included |
| Other lung conditions| | Respiratory system | Included |
| Stroke | Nervous system | Included |
| Epilepsy | Nervous system | Included |
| Migraine/Headaches | Nervous system | Included |
| Neurodegenerative diseases | Nervous system | Excluded |
| Neuropathy diseases | Nervous system | Excluded |
| Sleep disturbance | Nervous system | Included |
| Language and speech deficits | Nervous system | Included |
| Hearing problems | Special senses | Included |
| Vision impairment | Special senses | Included |
| Arthritis | Musculoskeletal system | Included |
| Back problems | Musculoskeletal system | Included |
| Osteoporosis | Musculoskeletal system | Excluded |
| Metabolic bone diseases | Musculoskeletal system | Excluded |
| Myopathy diseases | Musculoskeletal system | Excluded |
| Other musculoskeletal problems | Musculoskeletal system | Included |
| Falls | Functional | Included |
| Walking difficulties | Functional | Included |
| Upper limb dysfunction | Functional | Included |
| Restriction of physical activities | Functional | Included |
| Poor coordination | Functional | Excluded |
| Problems getting dressed | Functional | Excluded |
| Problems with bathing | Functional | Excluded |
| Problems with carrying out personal grooming | Functional | Excluded |
| Depression/Low Mood | Neuropsychiatric system | Included |
| Anxiety | Neuropsychiatric system | Included |
| Psychosis | Neuropsychiatric system | Excluded |
| Delirium | Neuropsychiatric system | Excluded |
| Learning disability | Neuropsychiatric system | Excluded |
| Memory impairment | Neuropsychiatric system | Excluded |
| Cognitive dysfunction | Neuropsychiatric system | Included |
| Other mental health problem | Neuropsychiatric system | Included |
| Kidney/urinary tract diseases | Genito-urinary system | Included |
| Bladder and sphincter control | Genito-urinary system | Included |
| Chronic kidney disease | Genito-urinary system | Excluded |
| Recurrent urinary tract infections/anatomical abnormalities | Genito-urinary system | Excluded |
| Reproductive diseases | Genito-urinary system | Excluded |
| Secondary sexual organ diseases (i.e. breast, prostate etc.) | Reproductive system | Excluded |
| Upper gastrointestinal problems | Digestive system | Included |
| Lower gastrointestinal diseases | Digestive system | Included |
| Liver diseases | Digestive system | Included |
| Gallbladder disease | Digestive system | Excluded |
| Pancreatic disease | Digestive system | Excluded |
| Lower gastrointestinal diseases | Digestive system | Included |
| Gastro-esophageal reflux disease | Digestive system | Excluded |
| Recurrent nausea and vomiting | Digestive system | Excluded |
| Dysphagia | Digestive system | Excluded |
| Inflammatory bowel disease | Digestive system | Excluded |
| Irritable bowel syndrome | Digestive system | Excluded |
| Persistent altered bowel habit | Digestive system | Excluded |
| Fecal continence | Digestive system | Excluded |
| Recurrent infections | Immune system | Excluded |
| Recurrent allergies | Immune system | Excluded |
| Hay fever | Immune system | Included |
| Eczema | Immune system | Excluded |
| Auto-immune disease | Immune system | Excluded |
| Immunizations | Immune system | Included |
| Skin diseases | Skin | Excluded |
| Cancer | Miscellaneous | Included |
| Genetic diseases | Miscellaneous | Excluded |
| Hernia | Miscellaneous | Included |
| Smoking | Miscellaneous | Included |
| Alcohol | Miscellaneous | Excluded |
| Shingles | Miscellaneous | Included |
| Hospital admissions | Miscellaneous | Excluded |
| Other diseases | Miscellaneous | Excluded |

*Inclusion status was established based on the availability of the data to allow for the same deficit to be appraised across the 4 time periods of the life-course.

**Supplementary Table S2**. 45-Item frailty index–brief explanation, scoring and the results from the first principal component weights.

| **Frailty index: Deficit** | **Deficit Scoring** | **PCA Weights at 60-64 years** |
| --- | --- | --- |
| **1. Congenital heart disease** | 0-Absent, 0.5-Partial deficit, 1- Present | 0.0031 |
| **2. Rheumatic heart disease** | 0-Absent, 0.5-Partial deficit, 1- Present | 0.0087 |
| **3. Hypertension** | 0-Absent, 0.5-Partial deficit, 1- Present | 0.0016 |
| **4. Ischaemic heart disease** | 0-Absent, 0.5-Partial deficit, 1- Present | 0.0267 |
| **5. Myocardial infarction*** | 0-Absent, 0.5-Partial deficit, 1- Present | 0.0145 |
| **6. Heart failure** | 0-Absent, 0.5-Partial deficit, 1- Present | 0.0066 |
| **7. Heart rhythm abnormality**† | 0-Absent, 0.5-Partial deficit, 1- Present | 0.0121 |
| **8. Other cardiovascular disease**‡ | 0-Absent, 0.5-Partial deficit, 1- Present | 0.0308 |
| **9. Anemia or coagulation defects**§ | 0-Absent, 0.5-Partial deficit, 1- Present | 0.0238 |
| **10. Cancer** | 0-Absent, 0.5-Partial deficit, 1- Present | 0.0073 |
| **11. Diabetes** | 0-Absent, 0.5-Partial deficit, 1- Present | 0.0141 |
| **12. Thyroid disease** | 0-Absent, 0.5-Partial deficit, 1- Present | 0.0191 |
| **13. Asthma** | 0-Absent, 0.5-Partial deficit, 1- Present | 0.0171 |
| **14. Respiratory Tract Infection** | 0-Absent, 0.5-Partial deficit, 1- Present | 0.0273 |
| **15. Respiratory symptoms**|| | 0-Absent, 0.5-Partial deficit, 1- Present | 0.0264 |
| **16. Other lung conditions** # | 0-Absent, 0.5-Partial deficit, 1- Present | 0.0023 |
| **17. Stroke** | 0-Absent, 0.5-Transient Ischaemic Attack, 1- Present | 0.0213 |
| **18. Epilepsy** | 0-Absent, 0.5-isolated fit, 1- Present | 0.0103 |
| **19. Migraine/Headaches** | 0-Absent, 0.5-Partial deficit, 1- Present | 0.0408 |
| **20. Sleep disturbance** | 0-Absent, 0.5-Partial deficit, 1- Present | 0.0267 |
| **21. Language and speech deficits** | 0-Absent, 0.5-Partial deficit, 1- Present | 0.0346 |
| **22. Hearing problems** | 0-Absent, 0.5-Partial deficit, 1- Present | 0.0165 |
| **23. Vision impairment** | 0-Absent, 0.5-Partial deficit, 1- Present | 0.0167 |
| **24. Fracture** | 0-Absent, 0.5-Partial deficit, 1- Present | 0.0191 |
| **25. Arthritis** | 0-Absent, 0.5-Partial deficit, 1- Present | 0.0326 |
| **26. Back problems** | 0-Absent, 0.5-Partial deficit, 1- Present | 0.0337 |
| **27. Other musculoskeletal problems**** | 0-Absent, 0.5-Partial deficit, 1- Present | 0.0024 |
| **28. Falls** | 0-Absent, 0.5-Partial deficit, 1- Present | 0.0504 |
| **29. Walking difficulties** | 0-Absent, 0.5-Partial deficit, 1- Present | 0.0585 |
| **30. Upper limb dysfunction**∳ | 0-Absent, 0.5-Partial deficit, 1- Present | 0.0448 |
| **31. Restriction of physical activities** | 0-Absent, 0.5-Partial deficit, 1- Present | 0.0595 |
| **32. Depression/Low Mood** | 0-Absent, 0.5-Partial deficit, 1- Present | 0.0439 |
| **33. Anxiety** | 0-Absent, 0.5-Partial deficit, 1- Present | 0.0349 |
| **34. Cognitive dysfunction**∅ | 0-Absent, 0.5-Partial deficit, 1- Present | 0.0230 |
| **35. Other mental health problems**〆 | 0-Absent, 0.5-Partial deficit, 1- Present | 0.0091 |
| **36. Kidney/urinary tract diseases** | 0-Absent, 0.5-Partial deficit, 1- Present | 0.0230 |
| **37. Bladder and sphincter control** | 0-Absent, 0.5-Partial deficit, 1- Present | 0.0365 |
| **38. Upper GI problems**ゝ | 0-Absent, 0.5-Partial deficit, 1- Present | 0.0288 |
| **39. Liver diseases** | 0-Absent, 0.5-Partial deficit, 1- Present | 0.0039 |
| **40. Lower GI diseases**✺ | 0-Absent, 0.5-Partial deficit, 1- Present | 0.0412 |
| **41. Hernia** | 0-Absent, 0.5-Partial deficit, 1- Present | 0.0087 |
| **42. Smoking** | 0-Absent, 0.5-Ex-smoker, 1- Current smoker or previous heavy smoker (>=15 pack-years) | 0.0141 |
| **43. Lack of immunizations~** | +0.25 per missed immunization (maximum 0.75) | 0.0058 |
| **44. Hay fever** | 0-Absent, 0.5-Partial deficit, 1- Present | 0.0146 |
| **45. Shingles** | 0-Absent, 0.5-Partial deficit, 1- Present | 0.0033 |

* Each subsequent myocardial infarct is granted another +0.5 up to a maximum of 2.

† Defined as atrioventricular or ventricular conduction defect or persistent palpitations or diagnosed arrhythmia or implanted pacemakers.

‡Defined as any other cardiovascular disease diagnosis such as cardiomyopathy, valve disease, etc.

§ Coagulation defects included both thrombophilia and bleeding disorders.

|| Defined as persistent shortness of breath, wheeze or sputum production in the absence of a chest infection.

# Defined as any other lung pathology, lung disease diagnosis or abnormal spirometry.

** Defined as any other musculoskeletal problem such as orthopedic defects, limb loss, abnormal DEXA bone mineral density etc.

∳ Defined as difficulties in picking up small objects, carrying larger objects, decreased grip strength or lower arm reach.

∅ Defined as learning difficulties or memory problems.

〆Defined as any other mental health problem including behaviour problems, aggression, delirium, hallucinations, mania etc.

ゝ Includes conditions such as gastro-oesophageal reflux disease, ulcers or indigestion

✺ Included conditions such as fecal incontinence, irritable bowel syndrome, inflammatory bowel diseases, persistent constipation or haemorrhoids.

~ Whooping cough, diphtheria or BCG.

*GI, gastrointestinal; PCA, principal component analysis.*

*.*

**Supplementary Table S3**. 45-Item frailty index–metadata used for scoring.

| **Frailty index: Deficit** | **Metadata and/or** **Metadata categories** |
| --- | --- |
| **1. Congenital heart disease** | Evidence of congenital heart disease 1957 and 1961 |
| **2. Rheumatic heart disease** | Any evidence of rheumatic heart disease 1957 and 1961 |
| **3. Hypertension** | How old were you then? [BP problem]  Whether SM's blood pressure is normal - WHO definition (140/90) (1982)  Have you had high blood pressure? (1989)  Has a doctor said you had this [BP] problem? (1999)  Hypertension or antihypertensive medication use at age 53  Hypertension BP>=140/90 - first measure (2009)  Hypertension BP>=140/90 – second measure (2009) |
| **4. Ischaemic heart disease** | How old were you when you first had this problem? [Angina]  Rose Angina questionnaires 1982 and 1989  Self-report of ever doctor diagnosed angina at 1999  Do you get it [pain or discomfort in chest] when you walk at an ordinary pace on the level? (2009)  Do you get this pain or discomfort [in your chest] when you walk uphill or hurry? (2009)  Does the pain or discomfort in your chest go away if you stand still? (2009) |
| **5. Myocardial infarction** | How old were you when you had your first heart attack?  Heart attack at 53 years  Which of these kinds of heart trouble [listed on card C] have you had, if any, in the last 10 years? (1999)  Self-report of ever doctor diagnosed MI at 1999  Have you ever suffered from a heart attack? 1999  How many heart attacks have you had? 1999  Since 1999 has a doctor told you that you have had a heart attack (myocardial infarct/coronary thrombosis)? (2009) |
| **6. Heart failure** | Illnesses for age period 0 - 60 months, categorised from ICD codes  Illnesses for age period 61 - 120 months, categorised from ICD codes  Illnesses for age period 121 - 180 months, categorised from ICD codes  Illnesses for age period 181 - 240 months, categorised from ICD codes  Illnesses for age period 241 - 300 months, categorised from ICD codes  Which of these kinds of heart trouble [listed on card C] have you had, if any, in the last 10 years? (1999)  Echocardiographic data (2009) |
| **7. Heart rhythm abnormality** | Heart rhythm lying 1952 and 1953  Heart rhythm standing 1952 and 1953  Illnesses for age period 181 - 240 months, categorised from ICD codes  Illnesses for age period 241 - 300 months, categorised from ICD codes  Which of these kinds of heart trouble [listed on card C] have you had, if any, in the last 10 years? (1999)  Palpitations - In the last 12 months have you had any of these symptoms and how much have they bothered you in everyday life? 1999 to 2000  Minnesota ECG groups (2009)  Do you have a pacemaker? (2009) |
| **8. Other cardiovascular disease** | Other heart abnormalities (those not mentioned in any other part of the form) 1952, 1953, 1957  Heart trouble 1952, 1953  Illnesses for age period 0 - 60 months, categorised from ICD codes  Illnesses for age period 61 - 120 months, categorised from ICD codes  Illnesses for age period 121 - 180 months, categorised from ICD codes  Illnesses for age period 181 - 240 months, categorised from ICD codes  Illnesses for age period 241 - 300 months, categorised from ICD codes  SM: heart trouble (1982)  Have you ever had heart trouble? (1989)  Which of these kinds of heart trouble [listed on card C] have you had, if any, in the last 10 years? (1999)  Has a doctor told you that you have other heart trouble? (2009)  Has a doctor told you that you have cardiomyopathy? (2009)  Has a doctor told you have aortic stenosis? (2009)  Since 1999 have you had any other heart trouble suspected or confirmed? (e.g. valve disease, congenital heart disease or irregular heart beat) (2009) |
| **9. Anaemia or coagulation defects** | Illnesses for age period 0 - 60 months, categorised from ICD codes  Illnesses for age period 61 - 120 months, categorised from ICD codes  Illnesses for age period 121 - 180 months, categorised from ICD codes  Illnesses for age period 181 - 240 months, categorised from ICD codes  Illnesses for age period 241 - 300 months, categorised from ICD codes  Use of Medications for anaemia (1989)  Have you had anaemia or any other blood disorder? (1989)  Specify. Anaemia or other blood disorder you suffer from (1989)  Have you taken any prescribed medicine for anaemia or any other blood disorder? (1989)  Check whether SM has clotting or bleeding disorder (1999)  Use of Medications for anaemia (2009)  Has your doctor told you that you have pulmonary embolism or systemic embolism within the last 4 weeks?  Has your doctor told you that you have a bleeding or clotting disorder? (2009)  Anaemia (moderate or severe) (2009) |
| **10. Cancer** | How old were you then? [first diagnosis of cancer]  SM: ever had cancer 1982,1989  Cancer at 53 years  Since 1999 have you been told by a doctor that you have cancer? (2009) |
| **11. Diabetes** | How old were you then? [Diagnosis of diabetes]  How old were you when you were first told you had diabetes?  Diabetes 1977, 1982, 1989  Diabetes at 53 years  Since 1999 have you been told that you have diabetes?  Diabetes diagnosed by GP, validation (2009) |
| **12. Thyroid disease** | How old were you then? [Diagnosis of thyroid problem]  Has a doctor said you had this [thyroid] problem? (1999)  Since 1999 have you been told by a doctor that you have a thyroid disorder? (2009) |
| **13. Asthma** | How old were you then? [Diagnosis of asthma]  Asthma 1957, 1961  Survey Member asthma 1982, 1989  Asthma at 53 years  Do you suffer from asthma or hay fever? (2009)  Since 2006, have you had asthma? |
| **14. Respiratory Tract Infection** | Pre-school lower respiratory infection (only 0-2 years)  Lower resp. infection 1952/53  One-week absences from school - summary upper respiratory infections 1952-56  One-week absences from school - summary bronchitis (1952-56)  SM: bronchitis? (1982)  Have you had bronchitis? (1989)  Whether SM has had any respiratory infections such as influenza, pneumonia etc., in past 3 weeks (1999)  During the past 3 years have you had any chest illness, for example, bronchitis or pneumonia, which has kept you off work or indoors for a week or more? (1999)  During the past 3 years have you had any chest illness, for example, bronchitis or pneumonia, which has kept you off work or indoors for a week or more? (2009)  In the past 3 weeks have you had any respiratory infections such as influenza, pneumonia, bronchitis or a severe cold? (2009) |
| **15. Respiratory symptoms** | Cough (without a cold) 1952 and 1953  Phlegm last 3 years (1966)  In the past three years have you had a period of cough and phlegm (spit from the chest) lasting for three weeks or more? 1971, 1982, 1989  Do you get short of breath walking with other people of your own age at an ordinary pace on the level? 1966, 1971  Does your chest ever sound wheezy or whistling? 1966, 1971  Cough for as much as 3 months 1966, 1982, 1989  Does your chest ever sound wheezy or whistling? 1999  Do you usually cough during the day or night in winter? 1999  In the past 3 years, have you had a period of cough and phlegm lasting for 3 weeks or more? 1999  Do you bring up phlegm on most days for as much as 3 months each year? 1999  Do you usually bring up any phlegm during the day or at night in winter? 1999  Respiratory symptoms at 60-64 (2009)  Do you usually bring up any phlegm (spit from the chest) first thing in the morning in winter? (2009)  Does your chest ever sound wheezy or whistling? (2009) |
| **16. Other lung conditions** | Lungs 1952, 1953, 1957, 1961  Illnesses for age period 0 - 60 months, categorised from ICD codes  Illnesses for age period 61 - 120 months, categorised from ICD codes  Illnesses for age period 121 - 180 months, categorised from ICD codes  Illnesses for age period 181 - 240 months, categorised from ICD codes  Illnesses for age period 241 - 300 months, categorised from ICD codes  First handicap, Second handicap, Third handicap (1972).  FEV1/FVC ratio (1999)  Respiratory disease (moderate or severe) (2009)  Respiratory disease (severe) (2009) |
| **17. Stroke** | How old were you then? [Diagnosis of stroke]  How many times have you consulted a doctor about a stroke in the last year? 1989  Have you had a stroke in the last 10 years? 1999  Has a doctor said you had this problem? [Stroke] 1999  Whether SM has been admitted to hospital for a heart complaint or stroke in the past six weeks. 1999  Stroke at 53 years  Have you had a sudden sight problem which got better after a day in the last 10 years? 1999  Have you had a sudden weakness in an arm or leg which got better after a day in the last 10 years? 1999  Since 1999 have you been told by a doctor that you have had a stroke? 2009  Self-report of doctor diagnosed stroke since 1999 |
| **18. Epilepsy** | Fits or convulsions 1952, 1953, 1957, 1961  Early or late onset of epilepsy from 1972 to 1982?  Epilepsy 1982, 1989  Epilepsy up to 1999  Epilepsy at 53 years  Have you ever had any fits or epilepsy in the last ten years? (1999)  Since 1999 have you been told by a doctor that you have fits or epilepsy? (2009)  Since 2006, have you had epilepsy? |
| **19. Migraine/Headaches** | How old were you then? [Diagnosis of migraine]  SM: headaches or migraine (1982)  Have you had severe headaches or migraine? (1989)  How many times have you consulted a doctor about severe headaches or migraine in the last year? (1989)  Frequent severe headaches/migraine - In the last 12 months have you had any of these symptoms and how much have they bothered you in everyday life? (2000) |
| **20. Sleep disturbance** | Nightmares or disturbed sleep during past year (asked at 6 years) Number of times any member of household has gone to child  Nightmares or disturbed sleep during past year (asked at 11 years)  I often have ideas running through my head so that I cannot sleep (1959)  Trouble with sleep (1977)  Trouble getting off to sleep in past month (1982)  Over the last year have you had trouble getting off to sleep? (1989)  Over the last year have you had trouble with waking up and not being able to get back to sleep? (1989)  Have you recently lost much sleep over worry? (1999)  GHQ: Lost much sleep over worry (2009)  Pittsburgh Sleep Quality Index (PSQI): total score (2009) |
| **21. Language and speech deficits** | Any speech abnormalities 1952, 1953, 1957, 1961  Because of your health do you have difficulty speaking? (1982)  Have you had a sudden speech problem which got better after a day in the last 10 years? (1999)  Since 1999 have you had a sudden speech problem which got better after a day? (2009)  In the last 12 months have you had a problem with finding the right word? (2009) |
| **22. Hearing problems** | Doctor's assessment of child's hearing - 1952, 1953, 1957, 1961  Hearing 1957  First handicap, Second handicap, Third handicap (1972)  Do you have great difficulty following a conversation if there is background noise, for example, a TV, radio or child playing (wearing your hearing aid)? (1989)  Note about hearing problem (1989)  Do you wear a hearing aid at all? (1989)  Whether SM had hearing difficulty during testing (1999)  Do you find it very difficult to follow a conversation if there is background noise? (without a hearing aid) (1999)  Do you wear a hearing aid? (1999)  In the last 12 months have you had a problem with hearing a normal conversation? (2009)  In the last 12 months have you had a problem with hearing conversation in a noisy room? (2009)  In the last 12 months have you had a problem with hearing over the phone? (2009) |
| **23. Vision impairment** | Squint summary 1952-61, 1965  Eye defects other than squint 1952, 1953, 1957, 1961  First handicap, Second handicap, Third handicap (1972)  What sight defects 1965  Whether SM had sight difficulty during testing 1999  Have you had cataracts, glaucoma or other serious eye trouble in the last ten years? 1999  Since 2006, have you had serious eye trouble such as cataracts, glaucoma or macular degeneration? |
| **24. Fracture** | Age of first break  Age Category of first broken bone  Have you broken a bone since you were 25 years old? (1999)  Since 2006 have you broken a bone?  Was the first break a late adult fracture (at or after 55 years)? |
| **25. Arthritis** | Illnesses for age period 0 - 60 months, categorised from ICD codes  Illnesses for age period 61 - 120 months, categorised from ICD codes  Illnesses for age period 121 - 180 months, categorised from ICD codes  Illnesses for age period 181 - 240 months, categorised from ICD codes  Illnesses for age period 241 - 300 months, categorised from ICD codes  Arthritis or rheumatism 1977, 1982, 1989  Knee and limb osteoarthritis at 53y  Hand osteoarthritis at 53y  Since 2006, have you had osteoarthritis?  Since 2006, have you had rheumatoid arthritis? |
| **26. Back problems** | Orthopedic defects 1961  SM sciatica, lumbago or recurring backache 1977, 1982, 1989  In the last 12 months have you had sciatica, lumbago or severe backache? 1999, 2009 |
| **27. Other musculoskeletal problems** | Orthopedic defects 1952, 1953, 1957, 1961  Other abnormalities of deformities 1953  First handicap, Second handicap, Third handicap (1972)  Loss of limb (1982)  Bone Mineral Density spine, total hip, femoral neck (2009) |
| **28. Falls** | I fall and trip over things (1959)  Due to long-term health problems or physical or mental disabilities do you fall or have difficulty keeping balance? (1989)  Do you easily fall or have difficulty keeping your balance because of long term health problems? (1999)  Easily fall or have difficulty keeping balance due to long term health problems? (2009) |
| **29. Walking difficulties** | Age of walking several steps without support  Do you have difficulty with walking without help caused by illness, injury or ill health? (1982)  Has your difficulty with walking without help lasted for the last 2 weeks? (1982)  Due to long-term health problems or physical or mental disabilities do you have difficulty walking up or down steps or stairs? (1989)  Due to long-term health problems or physical or mental disabilities do you have difficulty walking for a quarter of a mile on the level? (1989)  Do you find it difficult walking up and down stairs because of long term health problems? (1999)  Difficulty walking up and down stairs due to long term health problems? (2009)  Difficulty walking 1/4 mile on the level due to long term health problems? (2009)  Does your health now limit you in walking half a mile? If so how much (2009)  Does your health now limit you in walking one hundred yards? If so how much? (2009) |
| **30. Upper limb dysfunction** | Picking up a pencil with left/right hand (1961)  Picking up a penny with left/right hand (1961)  Picking up a pin with left/right hand (1961)  Picking up a matchstick with left/right hand (1961)  How difficult is it for you to put your left arm above your head to reach for something above you? (1989)  Using your left/right hand only can you pick up a small object, such as a safety pin? (1989)  Using your left/right hand only can you turn a tap on and off? (1989)  Using your left/right hand only can you pick up and carry a 5lb bag of potatoes? (1989)  Using your left/right hand only can you pick up and carry a pint of milk? (1989(  Due to long-term health problems or physical or mental disabilities do you have difficulty holding, gripping or turning things? (1989)  Left/right hand grip strength (1999)  Do you have difficulty because of long term health problems using either arm to reach up high above head or to reach behind to tuck a shirt in or do up a zip? (1999)  Maximum grip in dominant/non-dominant hand at age 60-64 (2009)  Are you able to pick up small objects, such as a safety pin? (2009)  Difficult to go shopping and carry a full bag of shopping in each hand due to long-term health problems? (2009) |
| **31. Restriction of physical activities** | Heart trouble-restriction of activities 1952, 1953, 1957, 1961  Due to long-term health problems or physical or mental disabilities is it difficult for you to get in and out of bed? (1989)  Can you get in and out of bed without help? (1989)  Due to long-term health problems or physical or mental disabilities is it difficult for you to get in and out of a chair? (1989)  Can you dress and undress without help? (1989)  Due to long-term health problems or physical or mental disabilities is it difficult for you to dress and undress? (1989)  Due to long-germ health problems or physical or mental disabilities is it difficult for you to use the toilet paper? (1989)  Difficult getting in and out of a chair due to long term health problem (2009)  Difficult getting in and out of bed due to long term health problems (2009)  Strawbridge Frailty domain 1: physical functioning (2009)  Difficult to go shopping and carry a full bag of shopping in each hand due to long-term health problems? (2009) |
| **32. Depression/Low Mood** | I often feel sad for no reason at all (1959)  I usually feel tired and worn out (1959)  Do you have frequent ups and downs in mood either with or without apparent cause? (1959)  Do you have frequent ups and downs in mood, either with or without apparent cause? (1972)  Do you keep reasonably cheerful or have you been very depressed or low-spirited recently? (1982)  SM: nervous or emotional trouble or persistent depression 1982, 1989  Have you recently been feeling run down and out of sorts? (1999)  Have you recently felt that life is entirely hopeless? (1999)  Have you recently felt that life isn't worth living? (1999)  Been feeling run down and out of sorts (2009)  Been thinking of yourself as a worthless person (2009)  Since 2006, have you had depression? |
| **33. Anxiety** | Anxious at 13/15 years - teacher´s opinion  Have there been times when you have been anxious or frightened? (1982)  Have you felt anxious, fearful or nervous for a considerable time in the last year? (1982)  Do you avoid situations which make you anxious? (1982)  Do you tend to get anxious in certain situations? e.g. travelling, being alone in a lift or a tube train. (1982)  Over the last year have you been in situations, such as in a crowd or an enclosed space or meeting people, when you became unduly anxious? (1989)  Have you recently been getting scared or panicky for no good reason? (1999)  Have you experienced anxiety or depression in the last 12 months and how much did it bother you? (1993-2000)  GHQ: Been getting scared or panicky for no good reason (2009) |
| **34. Cognitive dysfunction** | Concentration primary school 1956 - teacher's opinion (1956)  Concentration at 13 years  First handicap, Second handicap, Third handicap (1972)  Have you had any lapses of memory lately? (1982)  Total correct verbal (word-list) memory score (15 words, 3 trials, max 45 words) (1989)  Verbal memory total score at age 53 years (1999)  Over the past 12 months has SM had more trouble than used to with anything requiring memory e.g. keeping appointments etc? (1999)  Sum of 3-word list memory tests (2009)  Strawbridge Frailty domain 3: cognitive functioning (2009)  Since 2006, have you had memory problems? |
| **35. Other mental health problems** | Behaviour problems (1961)  Aggression at 15 years - teacher's opinion  Summary of teacher's assessment of nervous behaviour 1959, 1961  Have you ever had any nervous trouble? (1972)  Psychiatric summary - condensed (1982)  Over the past year have there been times when you heard or saw things that other people couldn't? (1999)  Over the past year have there been times when you felt that something strange was going on? (1999)  Over the past year have there been times when you felt people were against you? (1999)  Over the past year have you felt that your thoughts were directly interfered with or controlled by some outside force or person? (1999)  Over the past year have there been times when you felt very happy indeed without a break for days on end? (1999)  Since 2006, have you ever experienced delirium symptoms? |
| **36. Kidney/urinary tract diseases** | Illnesses for age period 0 - 60 months, categorised from ICD codes  Illnesses for age period 61 - 120 months, categorised from ICD codes  Illnesses for age period 121 - 180 months, categorised from ICD codes  Illnesses for age period 181 - 240 months, categorised from ICD codes  Illnesses for age period 241 - 300 months, categorised from ICD codes  SM kidney or bladder infection so that there is pain when passing water (1982)  Have you had kidney or bladder infections? (1989)  Have you had any kidney or bladder infections in the last 10 years? (1999)  Pain when passing urine - In the last 12 months have you had any of these symptoms and how much have they bothered you in everyday life? (2000)  Have you ever consulted a doctor or other health professional about urinary symptoms such as frequency (day or night), incontinence (losing urine), urgency or 'cystitis'? (2000)  Since 2006, have you had kidney disease?  Urine albumin (2009)  eGFR result code (2009) |
| **37. Bladder and sphincter control** | Age last wet bed up to 1961  Do you ever lose control of your bladder? (1989)  Do you ever have an urgent and strong desire to pass urine which is difficult to control? (1994-1999)  Do you leak urine when you make a physical effort, like lifting, running, coughing, sneezing or laughing? (1999)  Do you leak urine when you are asleep? (1999)  Do you leak urine without urgency or warning? (1999)  How often do you leak urine? |
| **38. Upper GI problems** | Illnesses for age period 0 - 60 months, categorised from ICD codes  Illnesses for age period 61 - 120 months, categorised from ICD codes  Illnesses for age period 121 - 180 months, categorised from ICD codes  Illnesses for age period 181 - 240 months, categorised from ICD codes  Illnesses for age period 241 - 300 months, categorised from ICD codes  SM: recurring stomach trouble (such as ulcers or acid indigestion) 1977, 1982, 1989  Have you had any stomach trouble such as ulcers, gastritis or acid indigestion in the last 10 years? (1999) |
| **39. Liver diseases** | Jaundice 1946 – 1957  Palpable masses 1957, 1961 e.g. liver, spleen  Illnesses for age period 0 - 60 months, categorised from ICD codes  Illnesses for age period 61 - 120 months, categorised from ICD codes  Illnesses for age period 121 - 180 months, categorised from ICD codes  Illnesses for age period 181 - 240 months, categorised from ICD codes  Illnesses for age period 241 - 300 months, categorised from ICD codes  Have you had a problem with the liver? (1989)  Have you had any liver disease in the last 10 years? (1999)  Liver disease (moderate or severe) (2009)  In the last twelve months, have you had recurring stomach trouble/indigestion? |
| **40. Lower GI diseases** | Illnesses for age period 0 - 60 months, categorised from ICD codes  Illnesses for age period 61 - 120 months, categorised from ICD codes  Illnesses for age period 121 - 180 months, categorised from ICD codes  Illnesses for age period 181 - 240 months, categorised from ICD codes  Illnesses for age period 241 - 300 months, categorised from ICD codes  Do you ever lose control of your bowels? (1989)  Have you had piles or haemorrhoids? (1989)  Have you had persistent constipation? (1989)  In the last 4 weeks have you had any of these symptoms - constipation? (1994-1999)  In the last 4 weeks have you had any of these symptoms: diarrhea and/or constipation? (1999)  Age when irritable bowel syndrome first diagnosed (1997)  In the last twelve months, have you had any other persistent problem with your bowels? |
| **41. Hernia** | Hernia or weakness in inguinal canal – 1952, 1953  Umbilical hernia 1957  Have you had a hernia? 1982, 1989  Have you had a hernia in the last 10 years? 1999 |
| **42. Smoking** | Age of starting to smoke (rep at 20 years)  Age of stopping smoking in years (rep at 20 years)  Smoking status at 43 years  Lifetime smoking pack years up to 43  Smoking status at 53 years  Smoking pack years 43-53 years  Smoking status at 64 years  Smoking pack years - period 53-63 years |
| **43. Lack of immunizations~** | Whooping cough immunization (1946-1952)  Diphtheria immunization (1948)  Has child had BCG Vaccination (1961) |
| **44. Hay fever** | How old were you then? [Diagnosis of hay fever]  Have you had hay fever in the last 10 years? (1999)  Do you suffer from asthma or hay fever? (2009) |
| **45. Shingles** | How old were you then? [Diagnosis of shingles] |

*BP, blood pressure; WHO, World Health Organization; SM, study member; ICD, International Classification of Disease; BCG, Bacillus Calmette-Guerin.*

**Supplementary Table S4.** Associations between the step-change in deficit accumulation with echocardiographic parameters (EF, LVmassi, MCFi and E/e’) at 60-64 years.

|  |  | **Model 1**  **(adjusted for sex)** | | **Model 2**  **(adjusted for sex + SEP)** | | **Model 3**  **(adjusted for sex + SEP + BMI)** | |
| --- | --- | --- | --- | --- | --- | --- | --- |
| **Echo Parameter*** | **FI** | ***β* (95% CI)** | ***p*-value** | ***β* (95% CI)** | ***p*-value** | ***β* (95% CI)** | ***p*-value** |
| **EF** | FI2**–**1 | -0.12 (-0.22, -0.01) | **0.028** | -0.12 (-0.22, -0.01) | **0.025** | -0.11 (-0.22, -0.01) | **0.032** |
|  | FI3**–**1 | -0.13 (-0.21, -0.04) | **0.005** | -0.13 (-0.22, -0.04) | **0.004** | -0.12 (-0.21, -0.03) | **0.007** |
|  | FI4**–**1 | -0.13 (-0.21, -0.06) | **<0.001** | -0.14 (-0.21, -0.06) | **<0.001** | -0.13 (-0.21, -0.06) | **0.001** |
|  | FI3**–**2 | -0.08 (-0.20, 0.04) | 0.210 | -0.08 (-0.20, 0.04) | 0.194 | -0.07 (-0.19, 0.05) | 0.224 |
|  | FI4**–**2 | -0.11 (-0.20, -0.01) | **0.025** | -0.11 (-0.21, -0.02) | **0.019** | -0.11 (-0.20, -0.01) | **0.027** |
|  | FI4**–**3 | -0.10 (-0.21, 0.02) | 0.115 | -0.10 (-0.22, 0.02) | 0.099 | -0.10 (-0.21, 0.02) | 0.117 |
| **LVmassi** | FI2**–**1 | 0.19 (-0.09, 0.47) | 0.183 | 0.17 (-0.11, 0.45) | 0.221 |  |  |
|  | FI3**–**1 | 0.20 (-0.04, 0.45) | 0.094 | 0.17 (-0.07, 0.41) | 0.157 |  |  |
|  | FI4**–**1 | 0.26 (0.06, 0.47) | **0.012** | 0.23 (0.03, 0.44) | **0.025** |  |  |
|  | FI3**–**2 | 0.12 (-0.21, 0.45) | 0.467 | 0.08 (-0.24, 0.41) | 0.618 |  |  |
|  | FI4**–**2 | 0.25 (-0.01, 0.50) | 0.063 | 0.21 (-0.04, 0.47) | 0.102 |  |  |
|  | FI4**–**3 | 0.27 (-0.05, 0.59) | 0.102 | 0.26 (-0.06, 0.58) | 0.115 |  |  |
| **MCFi** | FI2**–**1 | -0.23 (-0.62, 0.16) | 0.256 | -0.20 (-0.60, 0.19) | 0.310 |  |  |
|  | FI3**–**1 | -0.17 (-0.50, 0.16) | 0.312 | -0.13 (-0.46, 0.20) | 0.431 |  |  |
|  | FI4**–**1 | -0.27 (-0.55, 0.01) | 0.064 | -0.23 (-0.52, 0.06) | 0.113 |  |  |
|  | FI3**–**2 | -0.02 (-0.48, 0.43) | 0.922 | 0.02 (-0.44, 0.48) | 0.938 |  |  |
|  | FI4**–**2 | -0.24 (-0.60, 0.120 | 0.182 | -0.20 (-0.56, 0.17) | 0.276 |  |  |
|  | FI4**–**3 | -0.36 (-0.82, 0.10) | 0.116 | -0.33 (-0.79, 0.13) | 0.151 |  |  |
| **E/e’** | FI2**–**1 | -1.61 (-7.45, 3.99) | 0.581 | -1.60 (7.44, 4.00) | 0.584 | -2.25 (-8.23, 3.46) | 0.449 |
|  | FI3**–**1 | 2.71 (-2.04, 7.22) | 0.250 | 2.75 (-2.01, 7.28) | 0.245 | 1.84 (-3.04, 6.49) | 0.449 |
|  | FI4**–**1 | 5.39 (1.53, 9.13) | **0.006** | 5.44 (1.57, 9.17) | **0.005** | 4.55 (0.64, 8.33) | **0.020** |
|  | FI3**–**2 | 7.08 (0.71, 13.30) | **0.027** | 7.15 (0.75, 13.42) | **0.027** | 6.31 (-0.25, 12.74) | 0.056 |
|  | FI4**–**2 | 9.31 (4.64, 13.90) | **<0.0001** | 9.36 (4.69, 13.96) | **<0.0001** | 8.13 (3.48, 12.78) | **<0.001** |
|  | FI4**–**3 | 9.00 (2.74, 15.16) | **0.004** | 9.09 (2.81, 15.29) | **0.004** | 8.17 (1.99, 14.33) | **0.009** |

*All reported analyses here consisted of generalized linear models with gamma Distribution and log link except for E/e’ that used logistic regression.

Significant *p*-values are highlighted in bold.

*EF, ejection fraction; LVmassi, left ventricular mass indexed to body surface area; MCFi, myocardial contraction fraction indexed to body surface area; β*, regression coefficient; CI, confidence interval, *FI2–1, step change in deficit accumulation between FI19_44 and FI0­_16; FI3–1, step change in deficit accumulation between FI45_54 and FI0_16; FI4–1, step change in deficit accumulation between FI60_64 and FI0­_16; FI3–2, step change in deficit accumulation between FI45_54 and FI19_44; FI4–2, step change in deficit accumulation between FI60_64 and FI19_44; FI4–3, step change in deficit accumulation between FI60_64 and FI45_54; SEP, socio-economic-position; BMI, body mass index.*

**Supplementary Table S5.** Association between life-course frailty indices and LVEDVi at 60-64 years.

|  |  | **Model 1 (adjusted for sex)** | | **Model 2 (adjusted for sex +SEP)** | |
| --- | --- | --- | --- | --- | --- |
| **Echo Parameter*** | **Frailty index** | ***β* (95% CI)** | ***p*-value** | ***β* (95% CI)** | ***p*-value** |
| **LVEDVi** | FI0­_16 | -0.12 (-0.38, 0.14) | 0.361 | -0.12 (-0.38, 0.13) | 0.346 |
|  | FI19_44 | -0.03 (-0.21, 0.16) | 0.783 | -0.03 (-0.21, 0.16) | 0.759 |
|  | FI45_54 | -0.01 (-0.17, 0.14) | 0.869 | -0.02 (-0.17, 0.14) | 0.835 |
|  | FI60_64 | 0.04 (-0.09, 0.18) | 0.526 | 0.04 (-0.09, 0.18) | 0.555 |
|  | FI2–1 | 0.03 (-0.15, 0.22) | 0.709 | 0.03 (-0.15, 0.21) | 0.718 |
|  | FI3–1 | 0.03 (-0.12, 0.18) | 0.700 | 0.03 (-0.13, 0.18) | 0.718 |
|  | FI4–1 | 0.07 (-0.06, 0.21) | 0.271 | 0.07 (-0.06, 0.21) | 0.284 |
|  | FI3–2 | 0.01 (-0.20, 0.22) | 0.927 | 0.01 (-0.20, 0.22) | 0.944 |
|  | FI4–2 | 0.09 (-0.08, 0.25) | 0.303 | 0.08 (-0.08, 0.25) | 0.317 |
|  | FI4–3 | 0.13 (-0.08, 0.33) | 0.230 | 0.13 (-0.08, 0.33) | 0.237 |
|  | FIsum | -0.002 (-0.06, 0.05) | 0.941 | -0.003 (-0.06, 0.05) | 0.902 |
|  | FImean | -0.01 (-0.22, 0.20) | 0.941 | -0.01 (-0.23, 0.20) | 0.902 |

*Analysis used generalized linear models with gamma distribution and log link.

*A*bbreviations as in **Supplementary Table S4.**

**Supplementary Table S6.** Comparison between those excluded because of >20% missing data and those who did not having missing values.

|  | **Group with ≤ 20% missing data** | | **Group with >20% missing data** | | ***p*-value** |
| --- | --- | --- | --- | --- | --- |
|  | ***n*** | **Result** | ***n*** | **Result** |
| **EF, %** | 1217 | 64.41 ± 7.89 | 237 | 63.52 ± 7.67 | 0.105 |
| **LVmassi, g/m2** | 1006 | 114.91 ± 37.50 | 189 | 118.73 ± 46.98 | 0.293 |
| **MCFi** | 860 | 0.49 ± 0.19 | 160 | 0.50 ± 0.21 | 0.135 |
| **E/e’** | 1259 | 7.95 ± 2.13 | 238 | 7.84 ± 2.15 | 0.487 |

Data presented as mean ± standard deviation.

*A*bbreviations as in **Supplementary Table S4.**

**Supplementary Table S7.** Comparing those with complete echocardiographic variables (EF, LVmassi, MCFi, E/e’) *vs.* the whole group with the specified echocardiographic parameter present, *vs.* those with the specified echocardiographic parameter present but at least 1 other missing variable.

|  | **All 4 echo variables present** | | **Key echo variable present** | | ***p*-value*** | **Key echo variable present and at least 1 other missing variable**† | | ***p*-value*** |
| --- | --- | --- | --- | --- | --- | --- | --- | --- |
|  | ***n*** | **Result** | ***n*** | **Result** |  | ***n*** | **Result** |  |
| **EF, %** | 801 | 64. 74 ± 7.36 | 1217 | 64.41 ± 7.89 | 0.465 | 416 | 63.77 ± 8.81 | 0.108 |
| **LVmassi, g/m2** | 801 | 115.09 ± 38.28 | 1006 | 114.91 ± 37.50 | 0.997 | 205 | 114.01 ± 34.35 | 0.991 |
| **MCFi** | 801 | 0.50 ± 0.20 | 860 | 0.49 ± 0.19 | 0.514 | 59 | 0.41 ± 0.23 | **0.0008** |
| **E/e’** | 801 | 7.96 ± 2.10 | 1259 | 7.95 ± 2.13 | 0.751 | 458 | 7.91 ± 2.18 | 0.501 |

* *p*-value for differences *vs.* group with all 4 echocardiographic variables.

† Other than the key outcome echocardiographic variable for that row.

Data presented as mean ± standard deviation.

Significant *p*-values are highlighted in bold.

*A*bbreviations as in **Supplementary Table S4.**

**Supplementary Table S8.** Association between life-course frailty indices and echocardiographic parameters at 60-64 years after additional adjustment for socio-economic position in childhood.

| **Echo Parameter*** | **Frailty index** | ***β* (95% CI)** | ***p*-value** |
| --- | --- | --- | --- |
| **EF** | FI0­_16 | 0.03 (-0.12, 0.18) | 0.684 |
|  | FI19_44 | -0.10 (-0.21, 0.01) | 0.065 |
|  | FI45_54 | -0.11 (-0.20, -0.02) | **0.016** |
|  | FI60_64 | -0.12 (-0.20, -0.05) | **0.002** |
|  |  |  |  |
| **LVmassi** | FI0­_16 | 0.48 (0.09, 0.88) | **0.018** |
|  | FI19_44 | 0.40 (0.13, 0.68) | **0.005** |
|  | FI45_54 | 0.32 (0.08, 0.55) | **0.009** |
|  | FI60_64 | 0.33 (0.12, 0.53) | **0.002** |
|  |  |  |  |
| **MCFi** | FI0­_16 | -0.55 (-1.13, 0.04) | 0.065 |
|  | FI19_44 | -0.45 (-0.84, -0.06) | **0.026** |
|  | FI45_54 | -0.26 (-0.59, 0.06) | 0.121 |
|  | FI60_64 | -0.32 (-0.61, -0.04) | **0.028** |
|  |  |  |  |
| **E/e’** | FI0­_16 | 2.42 (-6.02, 10.60) | 0.568 |
|  | FI19_44 | -0.96 (-6.93, 4.69) | 0.746 |
|  | FI45_54 | 2.57 (-2.31, 7.23) | 0.289 |
|  | FI60_64 | 5.13 (1.26, 8.97) | **0.009** |

Significant *p*-values are highlighted in bold.

*All analyses used generalized linear models with gamma distribution and log link except for E/e’ that used logistic regression.

*A*bbreviations as in **Supplementary Table S4.**

**Supplementary Table S9**. Associations between frailty indices and echocardiographic parameters (EF, LVmassi, MCFi and E/e’) in the fully adjusted model after multiple imputation.

| **Echo Parameter*** | **Frailty index** | ***β* (95% CI)** | ***p*-value** |
| --- | --- | --- | --- |
| **EF** | FI0­_16 | 0.04 (-0.11, 0.19) | 0.628 |
|  | FI19_44 | -0.10 (-0.20, 0.01) | 0.069 |
|  | FI45_54 | -0.11 (-0.20, -0.03) | **0.008** |
|  | FI60_64 | -0.12 (-0.20, -0.05) | **0.002** |
|  | FIsum | -0.04 (-0.07, -0.01) | **0.009** |
|  | FImean | -0.16 (-0.28, -0.04) | **0.009** |
|  |  |  |  |
| **LVmassi** | FI0­_16 | 0.49 (0.06,0.91) | **0.026** |
|  | FI19_44 | 0.42 (0.16, 0.69) | **0.002** |
|  | FI45_54 | 0.35 (0.11, 0.58) | **0.004** |
|  | FI60_64 | 0.38 )0.16, 0.60) | **0.0007** |
|  | FIsum | 0.16 (0.08, 0.24) | **0.0002** |
|  | FImean | 0.62 (0.30, 0.94) | **0.0002** |
|  |  |  |  |
| **MCFi** | FI0­_16 | -0.39 (-1.05, 0.27) | 0.247 |
|  | FI19_44 | -0.46 (-0.86, -0.06) | **0.024** |
|  | FI45_54 | -0.34 (-0.70, 0.03) | 0.071 |
|  | FI60_64 | -0.41 (-0.73, -0.08) | **0.016** |
|  | FIsum | -0.16 (-0.28, -0.03) | **0.014** |
|  | FImean | -0.63 (-1.13, -0.13) | **0.014** |
|  |  |  |  |
| **E/e’** | FI0­_16 | 2.70 (-5.40, 10.80) | 0.511 |
|  | FI19_44 | -0.58 (-6.42, 5.27) | 0.847 |
|  | FI45_54 | 2.98 (-1.51, 7.47) | 0..192 |
|  | FI60_64 | 5.14 (1.55, 8.73) | **0.005** |
|  | FIsum | 1.30 (-0.23, 2.83) | 0.010 |
|  | FImean | 5.20 (-0.92, 11.32) | 0.010 |

Significant *p*-values are highlighted in bold.

*All analyses used generalized linear models with gamma distribution and log link except for E/e’ that used logistic regression.

*A*bbreviations as in **Supplementary Table S4.**

**Supplementary Table S10.** Association between FIs-cardio (life-course frailty indices after removing all cardiovascular-related deficits from within the frailty indices) and echocardiographic parameters at 60-64 years in the fully adjusted models

| **Echo Parameter*** | **Frailty index** | ***β* (95% CI)** | ***p*-value** |
| --- | --- | --- | --- |
| **EF** | FI0­_16-cardio | 0.02 (-0.10, 0.15) | 0.725 |
|  | FI19_44-cardio | -0.04 (-0.14, 0.05) | 0.351 |
|  | FI45_54-cardio | -0.04 (-0.12, 0.05) | 0.418 |
|  | FI60_64-cardio | -0.03 (-0.10, 0.04) | 0.379 |
|  |  |  |  |
| **LVmassi** | FI0­_16-cardio | 0.40 (0.07, 0.73) | **0.018** |
|  | FI19_44-cardio | 0.28 (0.04, 0.52) | **0.025** |
|  | FI45_54-cardio | 0.11 (-0.11, 0.32) | 0.317 |
|  | FI60_64-cardio | 0.13 (-0.05, 0.31) | 0.143 |
|  |  |  |  |
| **MCFi** | FI0­_16-cardio | -0.55 (-1.00, -0.03) | **0.036** |
|  | FI19_44-cardio | -0.41 (-0.75, -0.07) | **0.020** |
|  | FI45_54-cardio | -0.14 (-0.43, 0.16) | 0.373 |
|  | FI60_64-cardio | -0.20 (-0.45, 0.05) | 0.117 |
|  |  |  |  |
| **E/e’** | FI0­_16-cardio | 1.66 (-5.27, 8.46) | 0.635 |
|  | FI19_44-cardio | -1.90 (-7.25, 3.15) | 0.474 |
|  | FI45_54-cardio | -0.77 (-5.45, 3.63) | 0.740 |
|  | FI60_64-cardio | 1.08 (-2.58, 4.55) | 0.551 |

Significant *p*-values are highlighted in bold.

*All analyses used generalized linear models with gamma distribution and log link except for E/e’ that used logistic regression.

*EF, ejection fraction; LVmassi, left ventricular mass indexed by body surface area; MCFi, myocardial contraction fraction indexed by the body surface area.* Other abbreviations as in **Supplementary Table 4**.

**Supplementary Table S11.** Association between FIs-cardio (life-course frailty indices after removing all cardiovascular-related deficits from within the frailty indices) and echocardiographic parameters at 60-64 years after additional adjustment for socio-economic position in childhood.

| **Echo Parameter*** | **Frailty index** | ***β* (95% CI)** | ***p*-value** |
| --- | --- | --- | --- |
| **EF** | FI0­_16-cardio | 0.02 (-0.11, 0.15) | 0.771 |
|  | FI19_44-cardio | -0.05 (-0.14, 0.05) | 0.342 |
|  | FI45_54-cardio | -0.03 (-0.11, 0.05) | 0.468 |
|  | FI60_64-cardio | -0.03 (-0.10, 0.04) | 0.443 |
|  |  |  |  |
| **LVmassi** | FI0­_16-cardio | 0.33 (0.02, 0.67) | **0.046** |
|  | FI19_44-cardio | 0.27 (0.01, 0.52) | **0.039** |
|  | FI45_54-cardio | 0.13 (-0.09, 0.35) | 0.245 |
|  | FI60_64-cardio | 0.14 (-0.04, 0.33) | 0.137 |
|  |  |  |  |
| **MCFi** | FI0­_16-cardio | -0.46 (-0.94, -0.02) | **0.049** |
|  | FI19_44-cardio | -0.38 (-0.72, -0.04) | **0.032** |
|  | FI45_54-cardio | -0.08 (-0.38, 0.21) | 0.589 |
|  | FI60_64-cardio | -0.16 (-0.41, 0.10) | 0.224 |
|  |  |  |  |
| **E/e’** | FI0­_16-cardio | 1.19 (-5.97, 8.18) | 0.741 |
|  | FI19_44-cardio | -2.04 (-7.36, 2.98) | 0.439 |
|  | FI45_54-cardio | -1.06 (-5.77, 3.34) | 0.647 |
|  | FI60_64-cardio | 0.90 (-2.75, 4.37) | 0.618 |

Significant *p*-values are highlighted in bold.

*All analyses used generalized linear models with gamma distribution and log link except for E/e’ that used logistic regression.

*A*bbreviations as in **Supplementary Table S4**.

**Supplementary Table S12**. Associations between FIs-cardio (life-course frailty indices after removing all cardiovascular-related deficits from within the frailty indices) and echocardiographic parameters (EF, LVmassi, MCFi and E/e’) in the fully adjusted model after multiple imputation.

| **Echo Parameter*** | **Frailty index** | ***β* (95% CI)** | ***p*-value** |
| --- | --- | --- | --- |
| **EF** | FI0­_16-cardio | 0.03 (-0.10, 0.15) | 0.658 |
|  | FI19_44-cardio | -0.04 (-0.13, 0.05) | 0.392 |
|  | FI45_54-cardio | -0.03 (-0.11, 0.05) | 0.429 |
|  | FI60_64-cardio | -0.03 (-0.09, 0.04) | 0.430 |
|  | | | |
| **LVmassi** | FI0­_16-cardio | 0.37 (0.01, 0.73) | **0.049** |
|  | FI19_44-cardio | 0.28 (0.01, 0.55) | **0.047** |
|  | FI45_54-cardio | 0.12 (-10, 0.34) | 0.295 |
|  | FI60_64-cardio | 0.15 (-0.03, 0.33) | 0.104 |
|  | | | |
| **MCFi** | FI0­_16-cardio | -0.34 (-0.86, 1.19) | 0.209 |
|  | FI19_44-cardio | -0.40 (-0.77, -0.03) | **0.033** |
|  | FI45_54-cardio | -0.15 (-0.46, 0.16) | 0.340 |
|  | FI60_64-cardio | -0.23 (-0.50, 0.04) | 0.089 |
|  | | | |
| **E/e’** | FI0­_16-cardio | 2.16 (-4.38, 8.69) | 0.517 |
|  | FI19_44-cardio | -1.39 (-6.44, 3.65) | 0.588 |
|  | FI45_54-cardio | -0.38 (-4.86, 4.11) | 0.870 |
|  | FI60_64-cardio | 1.23 (-2.18, 4.66) | 0.479 |

Significant *p*-values are highlighted in bold.

*All analyses used generalized linear models with gamma distribution and log link except for E/e’ that used logistic regression.

*SE, standard error. A*bbreviations as in **Supplementary Table S4.**.

**Supplementary Table S13**. Associations between PCA-weighted frailty indices (*wFI*) and echocardiographic parameters (EF, LVmassi, MCFi and E/e’) in the fully adjusted models considering young adulthood and older age exemplars.

| **Echo Parameter*** | **Weighted Frailty index** | ***β* (95% CI)** | ***p*-value** |
| --- | --- | --- | --- |
| **EF** | *wFI* 19_44 | -0.004 (-0.05, 0.04) | 0.840 |
|  | *wFI* 60_64 | -0.05 (-0.11, 0.01) | 0.104 |
|  |  |  |  |
| **LVmassi** | *wFI* 19_44 | 0.13 (0.02, 0.24) | **0.018** |
|  | *wFI* 60_64 | 0.19 (0.04, 0.35) | **0.014** |
|  |  |  |  |
| **MCFi** | *wFI* 19_44 | -0.20 (-0.36, -0.05) | **0.012** |
|  | *wFI* 60_64 | -0.24 (-0.45, -0.02) | **0.033** |
|  |  |  |  |
| **E/e’** | *wFI* 19_44 | -1.10 (-3.52, 1.21) | 0.359 |
|  | *wFI* 60_64 | 1.63 (-1.38, 4.46) | 0.271 |

Significant *p*-values are highlighted in bold.

*All analyses used generalized linear models with gamma distribution and log link except for E/e’ that used logistic regression.

*A*bbreviations as in **Supplementary Table S4**

**Supplementary Table S14**. Associations between frailty index and echocardiographic parameters (EF, LVmassi, MCFi and E/e’) in the fully adjusted model in random coefficients generalized linear mixed models.

| **Echo Parameter*** | **Frailty index** | ***β* (95% CI)** | ***p*-value** |
| --- | --- | --- | --- |
| **EF** | FI | -0.06 (-0.11, -0.01) | **0.017** |
| **LVmassi** | FI | 0.25 (0.14, 0.36) | **<0.0001** |
| **MCFi** | FI | -0.25 (-0.41, -0.09) | **0.002** |
| **E/e’** | FI | 1.94 (-0.09, 3.97) | 0.061 |

Significant *p*-values are highlighted in bold.

*All analyses used random coefficients generalized linear mixed models (glmm) with gamma distribution and log link except for E/e’ that used glmm with binomial distribution.

*A*bbreviations as in **Supplementary Table S4.**

**Supplementary Figure S1.** Life-course frailty index patterns of participants that had <20% missing data. Raw frailty indices (y-axis) at the 4 time periods are vertically represented for NSHD study members (x-axis).

*FI0_16, frailty index at 0 to 16 years; FI19_44, frailty index at 19 to 44 years; FI45_54, frailty index at 45-54 years; FI60_64, frailty index at 60-64 years.*


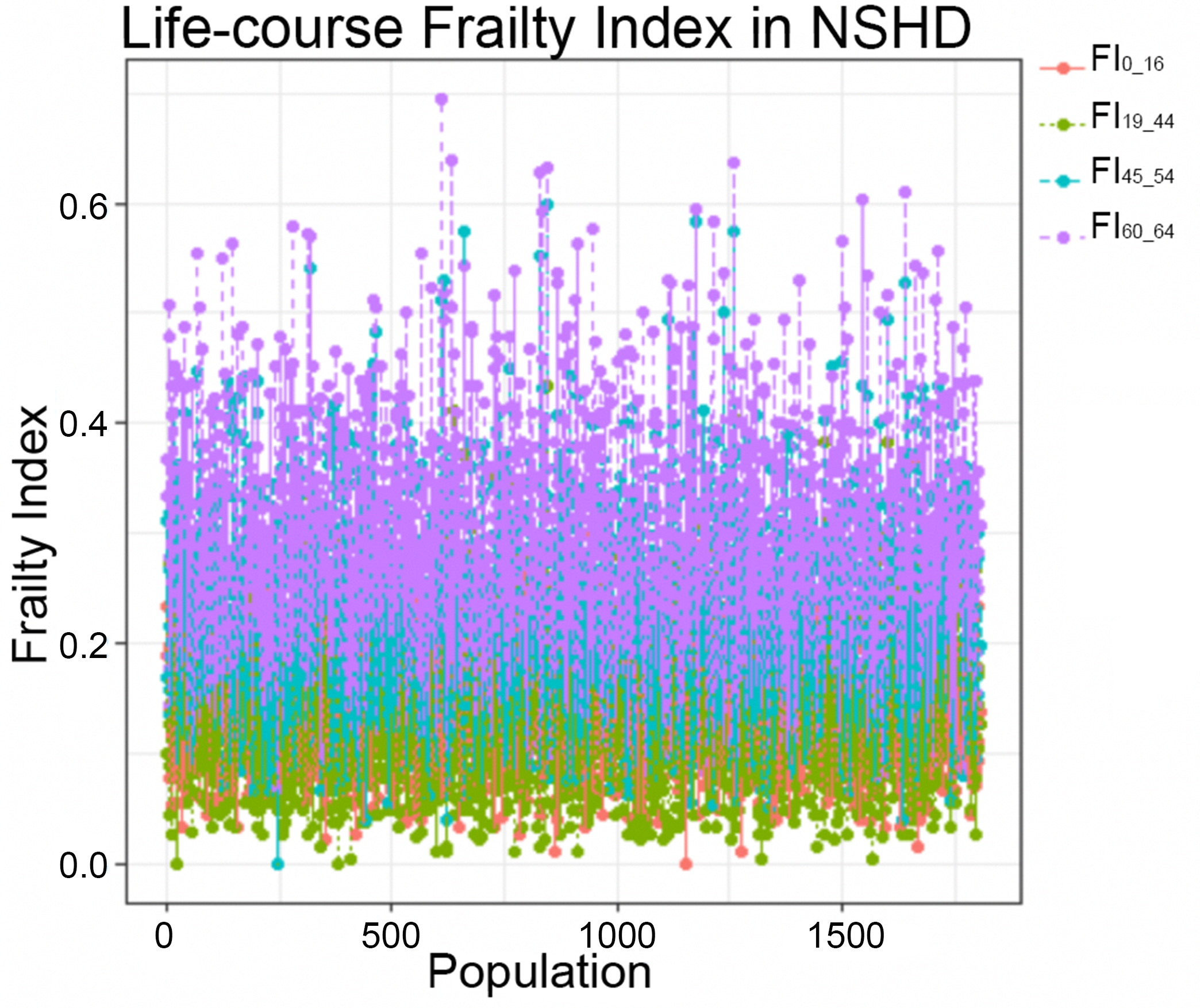


**Supplementary Figure S2.** Bar chart illustrating the frailty contributors (y-axis) in NSHD participants with less <20% missing data across the life-course: (**A**) 0-16 years; (**B**) 19-44 years; (**C**) 45-54 years; (**D**) 60-64 years.

*GI, gastrointestinal.*


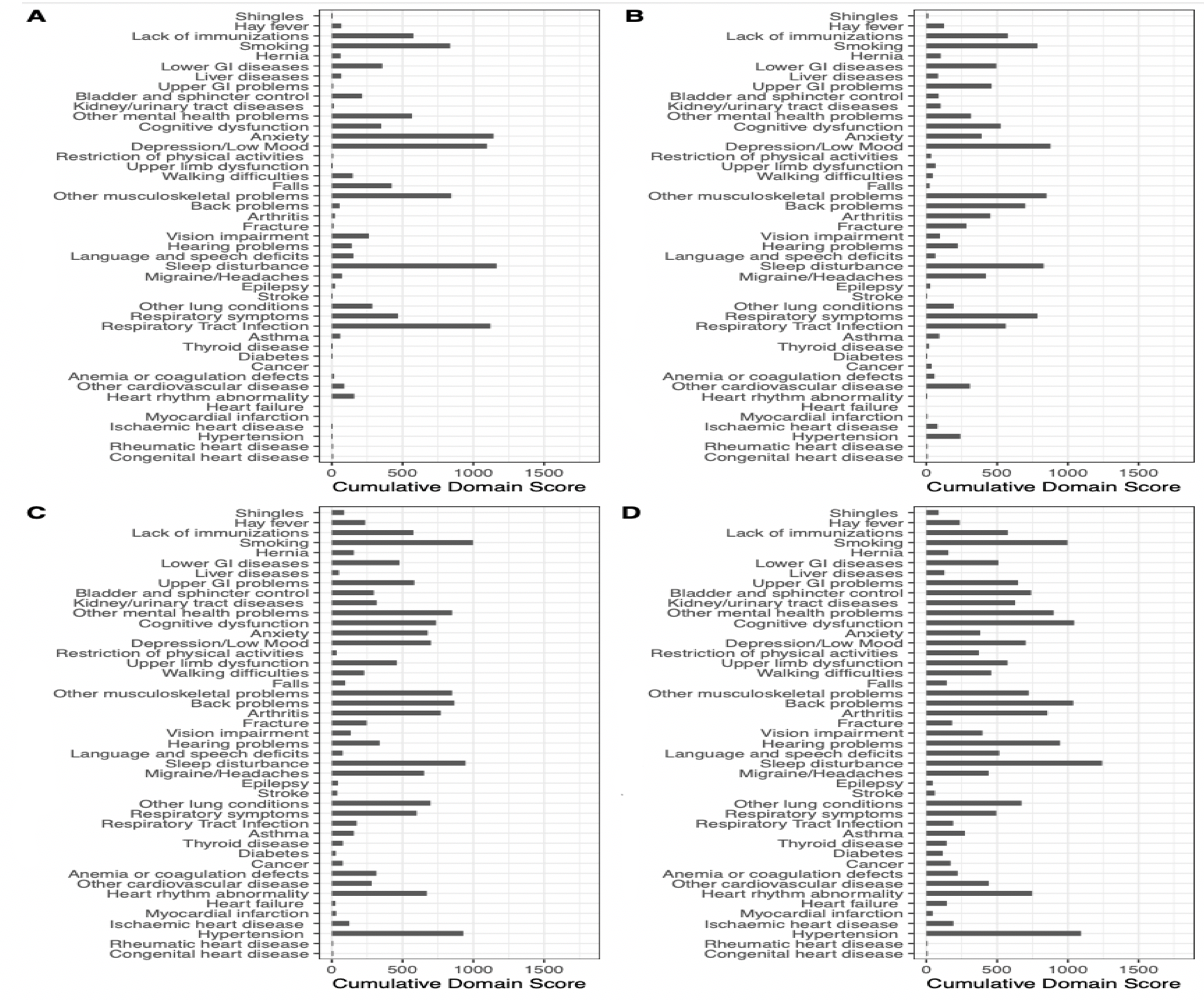


**Supplementary Figure S3**. Cluster plot based on the 45-health deficits at 0-16 years in NSHD participants.

*The cluster analysis revealed no clear separation of participants in terms of health deficit patterns at 0 to 16 years old time-period.*


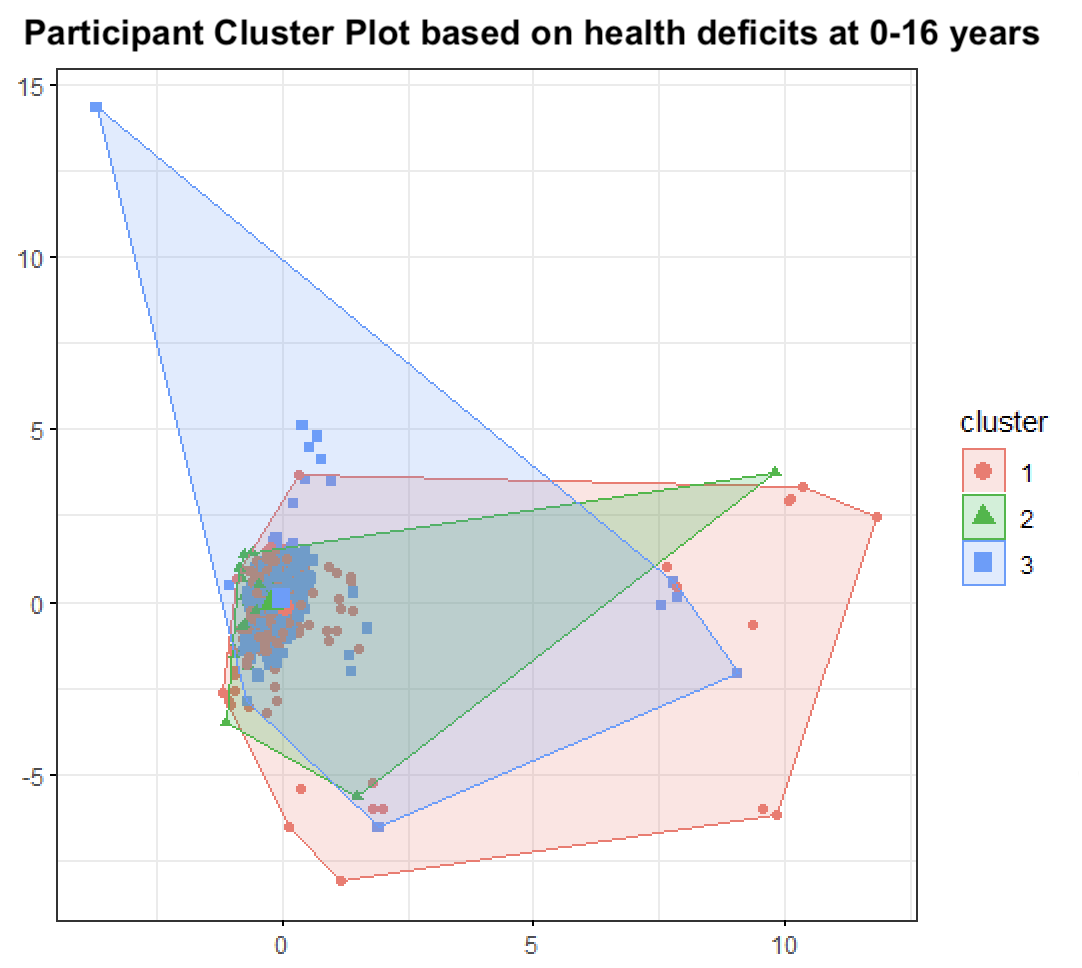
\

**Supplementary Figure S4**. Cluster plot based on the 45-health deficits at 19-44 years in NSHD participants.

*The cluster analysis revealed no clear separation of participants in terms of health deficit patterns at 19 to 44 years old time-period.*

**
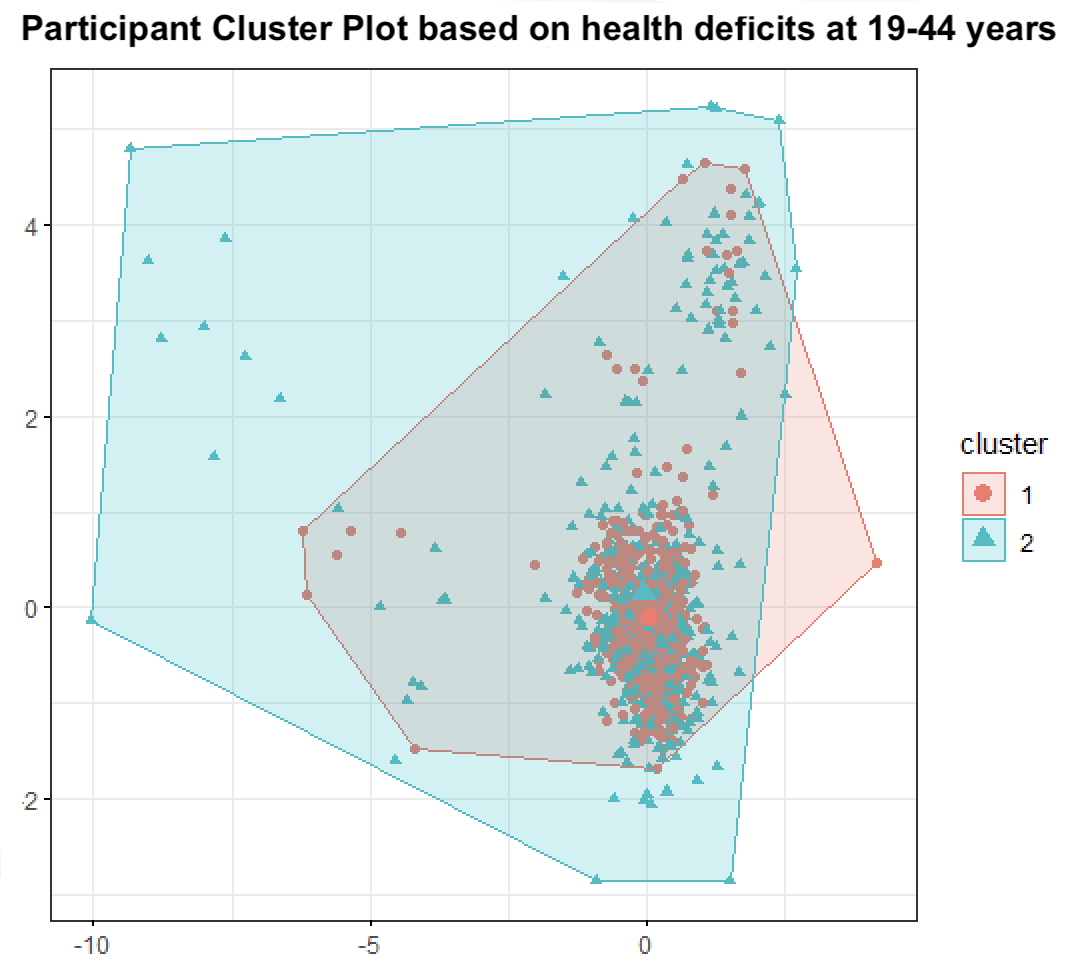
**

**Supplementary Figure S5** Cluster plot based on the 45-health deficits at 45-54 years in NSHD participants.

*The cluster analysis revealed no clear separation of participants in terms of health deficit patterns at 45 to 54 years old time-period.*


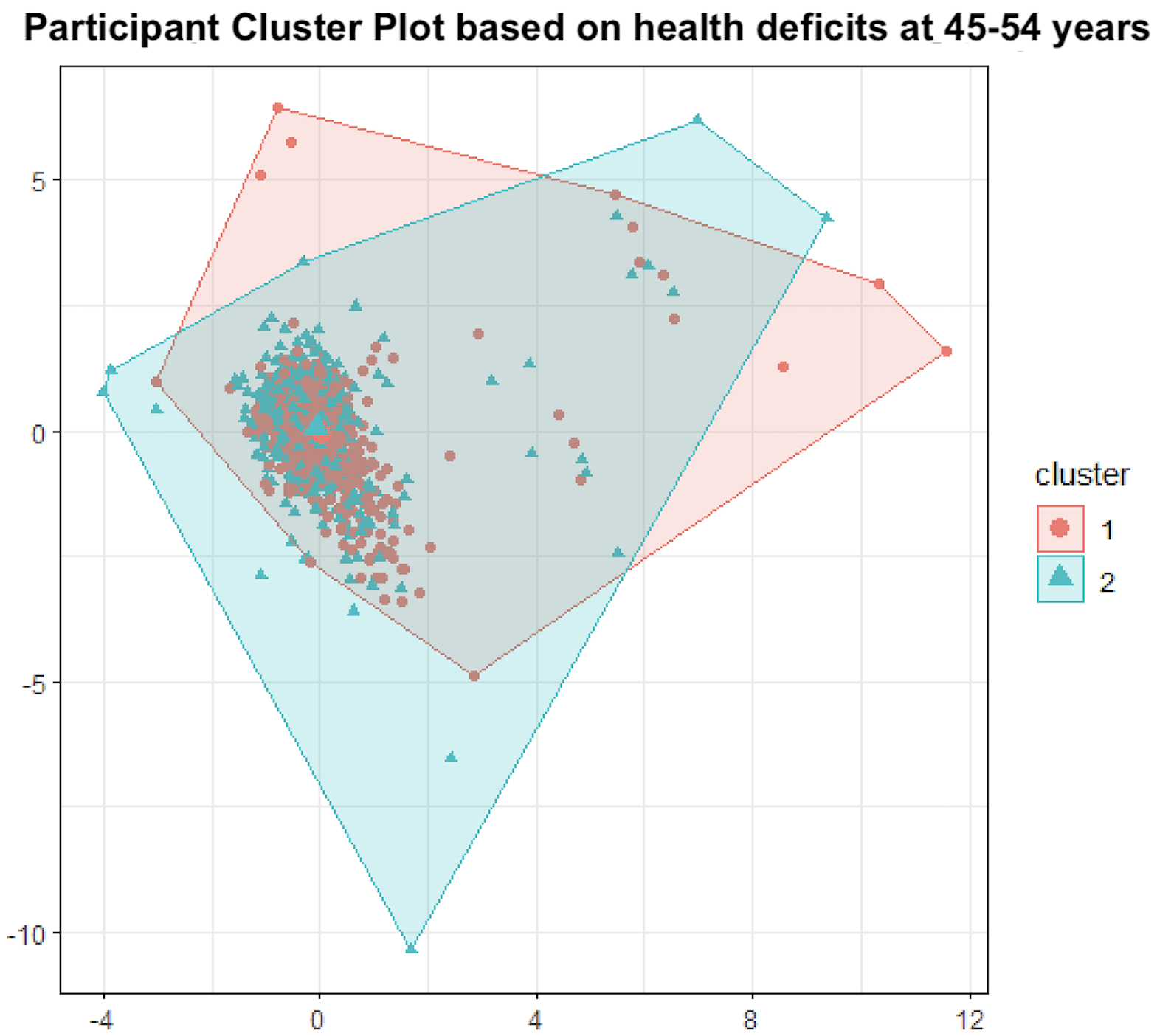


**Supplementary Figure S6**. Cluster plot based on the 45-health deficits at 60-64 years in NSHD participants.

*The cluster analysis revealed no clear separation of participants in terms of health deficit patterns at 60 to 64 years old time-period.*


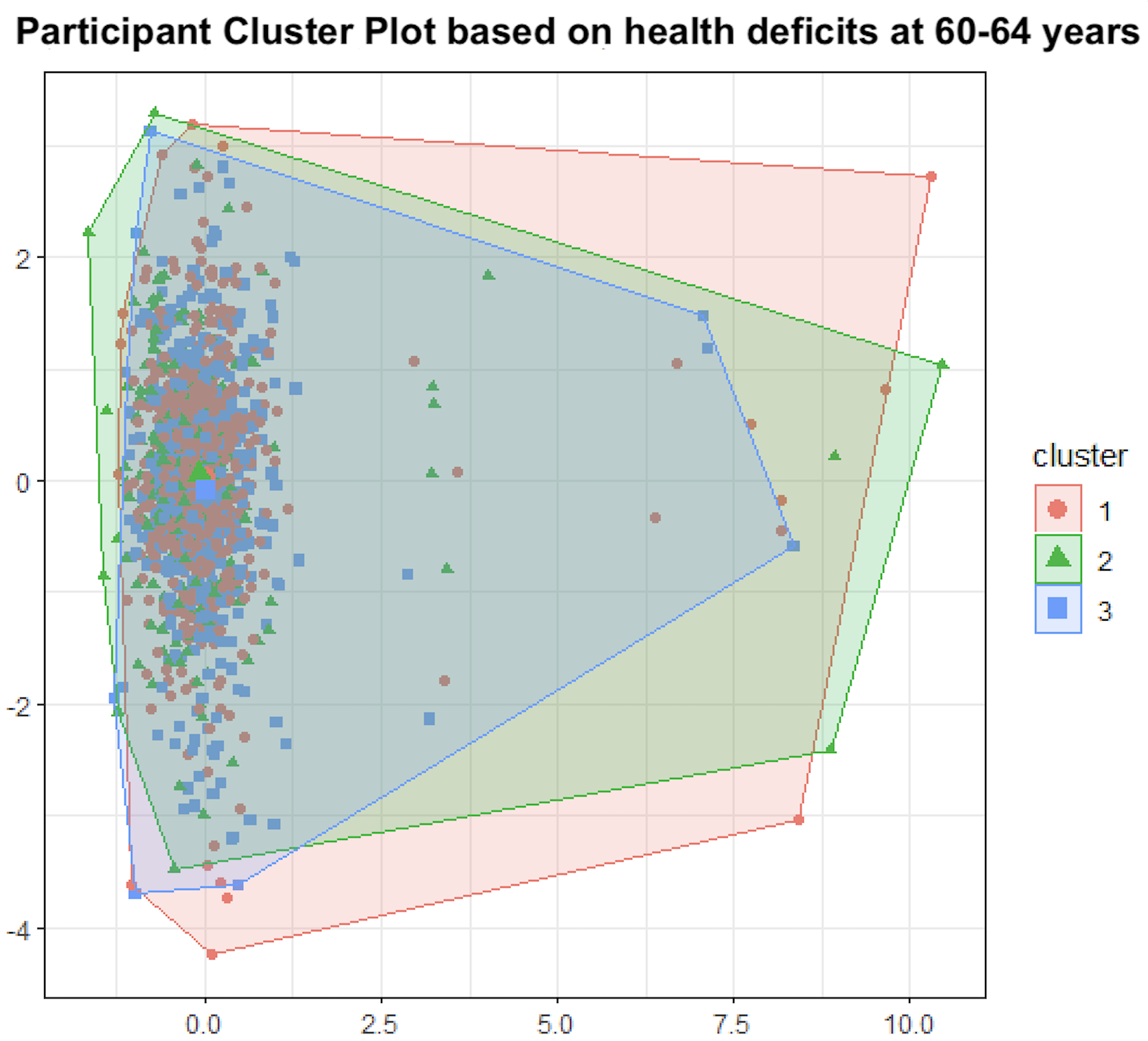


**Supplementary Figure S7.** 4-Dimensional plot showing the relationship between overall-life burden of health deficits (FImean) in NSHD participants and older age cardiac function in terms of MCFi (z-axis), LV EF (y-axis) and E/e’ (x-axis).  Health deficit categories for FImean have been defined as follows: green = FI >1SD below the mean; yellow = FI <1SD below the mean; orange = FI <1SD above the mean; red = FI >1SD above the mean.

*FImean, mean of FI0­_16, FI19_44, FI45_54 and FI60_64*; *MCFi, myocardial contraction fraction index; EF, ejection fraction.*


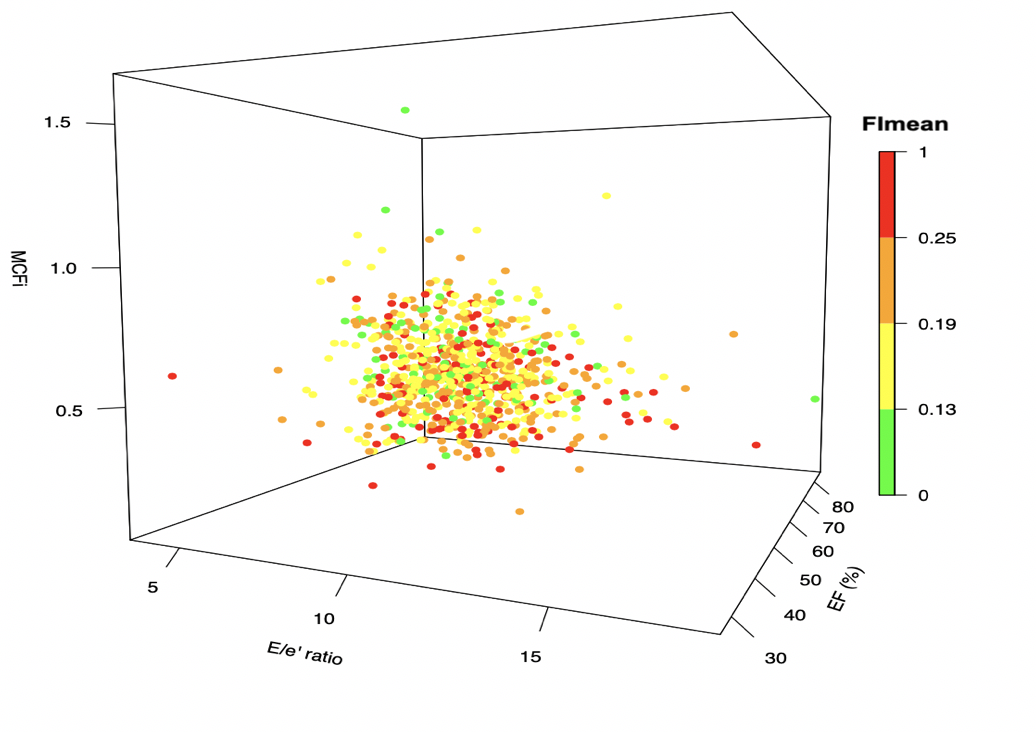

Supplement: Supplementary file 1 — Supplementary Information. [file 41598_2021_85435_MOESM1_ESM.doc]
